# Evolutionary analysis of the ENTH/ANTH/VHS protein superfamily reveals a coevolution between membrane trafficking and metabolism

**DOI:** 10.1186/1471-2164-13-297

**Published:** 2012-07-02

**Authors:** Johan-Owen De Craene, Raymond Ripp, Odile Lecompte, Julie D Thompson, Olivier Poch, Sylvie Friant

**Affiliations:** 1 Department of Molecular and Cellular Genetics, UMR7156 CNRS/Université de Strasbourg, 21 rue Descartes, 67084, Strasbourg, France; 2 Integrative Bioinformatics and Genomics, Institut de Génétique et de Biologie Moléculaire et Cellulaire IGBMC (CNRS/INSERM/UdS), 1 rue Laurent Fries, 67404, Illkirch, France

**Keywords:** Membrane trafficking, Cytokinesis, Metabolism, Comparative genomic, Eukaryotic evolution, Phylogeny

## Abstract

**Background:**

Membrane trafficking involves the complex regulation of proteins and lipids intracellular localization and is required for metabolic uptake, cell growth and development. Different trafficking pathways passing through the endosomes are coordinated by the ENTH/ANTH/VHS adaptor protein superfamily. The endosomes are crucial for eukaryotes since the acquisition of the endomembrane system was a central process in eukaryogenesis.

**Results:**

Our *in silico* analysis of this ENTH/ANTH/VHS superfamily, consisting of proteins gathered from 84 complete genomes representative of the different eukaryotic taxa, revealed that genomic distribution of this superfamily allows to discriminate Fungi and Metazoa from Plantae and Protists. Next, in a four way genome wide comparison, we showed that this discriminative feature is observed not only for other membrane trafficking effectors, but also for proteins involved in metabolism and in cytokinesis, suggesting that metabolism, cytokinesis and intracellular trafficking pathways co-evolved. Moreover, some of the proteins identified were implicated in multiple functions, in either trafficking and metabolism or trafficking and cytokinesis, suggesting that membrane trafficking is central to this co-evolution process.

**Conclusions:**

Our study suggests that membrane trafficking and compartmentalization were not only key features for the emergence of eukaryotic cells but also drove the separation of the eukaryotes in the different taxa.

## Background

Intracellular compartments represented by membrane-delineated regions with specific lipids and proteins contents are characteristic of eukaryotic cells. Membrane trafficking connects all compartments allowing on the one hand lipids and proteins synthesized in the endoplasmic reticulum (ER) to reach their intended organelle and on the other hand exchanges with the extracellular medium [1]. This makes membrane trafficking an important object of evolutionary studies, since these mechanisms are fundamentally inherent to the multi-organelle status of eukaryotes [2,3]. Among the different intracellular compartments, endosomes are central, since they are at the crossroads of several trafficking pathways and should therefore contain the vestiges of the first eukaryotic endomembrane system, a key factor for later evolution [4]. Endosomes are at the intersection of the endocytic, phagocytic, Golgi to lysosome trafficking (also termed VPS for vacuolar protein sorting), autophagy and plasma membrane recycling pathways. At the endosomes, effectors and cargo proteins following these different pathways are sorted to reach their final destination, henceforth we will gather this under the name endosomal system [5]. Specific key regulators of the endosomal system contain an ENTH (Epsin N-terminal homology), an ANTH (AP180 N-terminal homology) or a VHS (Vps27, Hrs and STAM) domain at their N-terminus [6]. Structural analyses resulted in the grouping of these proteins in the ENTH/ANTH/VHS superfamily, while functional analyses and sequence homologies led to their further classification into families and subfamilies [7-10].

These regulators also termed adaptors localize at the Golgi, endosomal or plasma membrane and function in pairs of proteins from different subfamilies such as the ANTH Sla2/HIP1 and the ENTH Ent1-2/Epsin1,2,3 in endocytosis. They are required for cargo sorting into vesicles and recruitment of scaffold or accessory trafficking effectors [10]. Indeed, most ENTH and ANTH domains are lipid binding domains specifically interacting with a given phosphoinositide enriched at the target membrane [6]. Cargo recruitment is mediated by interaction either with ubiquitin tagging the cargo (for endocytosis and late endosomal/multivesicular body (MVB) sorting or a peptide-motif as for the mannose-6-phosphate receptor (for transport of soluble lysosomal enzymes to endosomes) [11,12]. Moreover, to ensure the correct assembly and budding of vesicles, most plasma membrane and Golgi localized adaptors also interact with clathrin through their C-terminal clathrin binding sites [12,13]. Indeed, clathrin, the major component of vesicle coats at the plasma membrane and Golgi, depends on such adaptors for its membrane recruitment.

In addition to being studied experimentally, many protein families involved in trafficking have been analyzed in evolutionary studies [2,3]. For example, extensive studies of the COP (coat protein complex) proteins required for the formations of the vesicles at early secretory pathway (ER, Golgi trafficking) showed that they have a similar structure to clathrin coat, suggesting an evolutionary conservation of the vesicular coat structure [14,15]. Other in-depth investigations include the GTPase (Rab) and SNARE (SNAP (Soluble NSF Attachment Protein) REceptor) families that are required for the formation of the trafficking vesicles and their fusion with membranes ( [4,16]. All these studies highlighted the determinant role played by these proteins in the evolution of the endomembrane system. On the other hand, Field and co-workers focused on assessing the presence/absence profile of trafficking proteins belonging to many families using two representative organisms of each eukaryotic taxa [17]. They showed that in the endosomal system some effectors arose later than others during evolution, Vps27/Hrs and Hse1/STAM (VHS proteins) forming the ESCRT-0 (endosomal sorting complex required for transport) complex or the GGA proteins required for exit from the Golgi (VHS proteins) or the plasma membrane epsins (ENTH proteins) are specific to Fungi and Metazoa (Opisthokonta) [17,18].

Here we performed an extensive analysis of ENTH/ANTH/VHS superfamily members found in the proteomes of 84 fully sequenced organisms representing different eukaryotic taxa. Analysis of the presence/absence profiles of the different subfamilies revealed that the ENTHA, PICALM and GGA subfamilies were present prior to the divergence in the various eukaryotic taxa. Moreover, the genomic distribution of this superfamily perfectly reflects the dichotomy between Opisthokonta and Plantae. Comparative genomics of four proteomes and phylogenetic analyses revealed that a similar dichotomy was also observed for other protein families involved in the endosomal system but also in cytokinesis and metabolism. Further analyses of these protein families show that the endosomal system was a key process linking both metabolism and cytokinesis. Based on these results, we suggest an innovative evolutionary scenario, where the endosomal system drove the separation of Fungi and Metazoa from Plantae and Protists.

## Results

### Clustering of the ENTH/ANTH/VHS protein sequences

We analyzed the protein sequences of ENTH/ANTH/VHS superfamily members found in the genomes and proteomes of 84 fully sequenced Metazoa, Fungi, Amoebazoa, Plantae, Excavata, Euglenozoa, Chromista and Rhizaria taxa (Genome OnLine Database website November 2011). Proteins with an ANTH, ENTH or VHS domain were gathered by extensive BLAST (BLASTP, TBLASTN and PSI-BLAST) searches and used to generate a Multiple Alignment of Complete Sequences (MACS) composed of 1134 proteins manually adjusted according to the structural data available on the distinct ENTH, ANTH and VHS proteins. At least one protein was found in all organisms except for one Fungi (*E. cuniculi*) and three Chromista (*H. parasitica*, *T. pseudonana* and *C. hominis*). Analysis of the MACS allowed the clustering of all the proteins in: i) 4 VHS subfamilies GGA (Golgi-localized, gamma-ear-containing, ARF-binding proteins), STAM (signal transducing adaptor molecule (SH3 domain and ITAM motif)), VHS (Vps27/Hrs/STAM) and TOM (target of myb1), ii) 2 ANTH subfamilies ANTH and PICALM (Phosphatidylinositol binding clathrin assembly protein), and iii) 4 ENTH subfamilies Epsin, EpsinR and two newly identified subfamilies of unknown function. To facilitate the discussion, we named the Golgi/endosome EpsinR subfamily: ENTHA, the endocytic Epsin subfamily: ENTHB, the new vertebrate specific epsins (human ENTD1): ENTHC and the new fungi specific epsins (*S. cerevisiae* Ent4): ENTHD.

**Figure 1:**
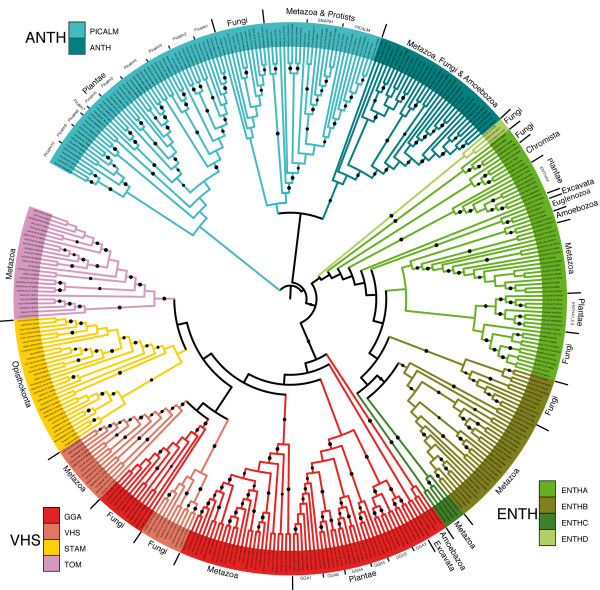
** Phylogeny of the proteins with an ENTH, ANTH or VHS domain.** Unrooted tree displaying the grouping of most ANTH, ENTH and VHS proteins from 42 fully sequenced organisms. The tree was generated using the highly conserved ∝-helices 2 to 7 of the N-terminal domain. (●) indicates branches with at least 70% confidence after 500 bootstrap calculations. Protein subfamilies are indicated by different colors.

As the C-terminal parts of these proteins are highly divergent and frequently specific of each family or subfamily, a robust phylogenetic tree (see Material and Methods section) was calculated using the most conserved common region of the aligned proteins (between ∝-helices 2 and 7 of the N-terminal domain). The phylogenetic tree (Figure 1) based on the conserved N-terminal domain shows a very similar clustering to the one obtained with the full-length proteins thus suggesting that the N- and C-terminal parts of ENTH/ANTH/VHS proteins have experienced concerted selective pressures during evolution. Broadly, Plantae proteins of the ANTH family (PICALM, ANTH subfamilies) and the VHS family (GGA, VHS, STAM, TOM subfamilies) clustered on a separate branch from Opisthokonta proteins. The ENTH subfamilies did not display such separation even though our phylogenetic tree confirmed that ENTHA-containing proteins were localized on a separate branch from the ENTHB-containing protein (Figure 1) as previously described [17]. Indeed, most Plantae ENTHA subfamilies (ENTHA1,2,3) clustered among the Opisthokonta group and the last one (ENTHA4) clustered on a separate branch displaying a closer relationship with protist and some fungal proteins (ENTHC). Interestingly, the yeast Ent5 protein and its fungal homologues are members of the ENTH family. Due to its sequence divergence, the yeast Ent5 protein was described either as belonging to the ENTH or ANTH family [19,20]. Based on our extended MACS and the phylogenetic tree, it clearly stands out that this small group is clearly distinct from all other Fungal ANTH members which cluster together on one branch separated from ENTH members. This is in keeping with the *in vivo* function of the yeast Ent5 in Golgi and endosomal sorting, a function performed by members of the ENTHA subfamily [19,20]. 

Finally, an important point results from the analysis of the Plantae, Euglenozoa and Amoebozoa proteins belonging to the VHS family. While our phylogenetic tree clustered these proteins with the opisthokonta GGA subfamily, previous studies have classified them in the TOM subfamily due to a domain organization similar to the metazoan TOM, i.e. presence of a GAT (GGA and Tom1) domain and absence of a GAE (gamma-adaptin ear) domain [21]‐ [23]. To definitively assign these proteins to either the GGA or the TOM subfamily, we aligned their GAT domain and a robust phylogenetic tree was calculated (Additional file 1: Figure S1). This tree showed a clear clustering of all Plantae and protist GAT domains on the same branch as Metazoa and Fungi GGA proteins whereas the Metazoan GAT domain of TOM proteins clustered on a separate branch. Confidence in this classification was acquired by analyzing both our alignment and the crystal structures of these domains. Our alignment showed that the extreme N-terminus of TOM proteins had a conserved PF domain and an extra amino acid between ∝-helices 3 and 4 compared to Metazoa GGA proteins or the Plantae and Protists proteins with a VHS domain (Additional file 2: Figure S2). Furthermore, the resolved structure of TOM1 VHS domain shows that the conserved PF domain is a ∝-helical turn and ∝-helix 6 of GGA is broken in TOM proteins [24,25]. These results clearly classify Amoebozoa, Plantae and Euglenozoa VHS proteins in the GGA subfamily and suggest that the GAE domain was a later acquisition of Opisthokonta GGA proteins.

As a whole, GGA-containing proteins revealed a complex evolution as illustrated by the fungal GGA branch which is nested in the VHS subfamily cluster and closer to the Metazoa than Fungi VHS domain (Figure 1). Since Fungal GGA proteins display the same domain composition and function as the Mammalian ones [26], this result suggests that the VHS domain, specific to the Opisthokonta taxa, evolved from the GGA domain and certainly resulted from duplication. Furthermore, the STAM and TOM subfamily, respectively specific to Opisthokonta and Metazoa, are more recent acquisitions that evolved from the VHS subfamily.

### Genomic distribution of the ENTH/ANTH/VHS superfamily members

**Figure 2:**
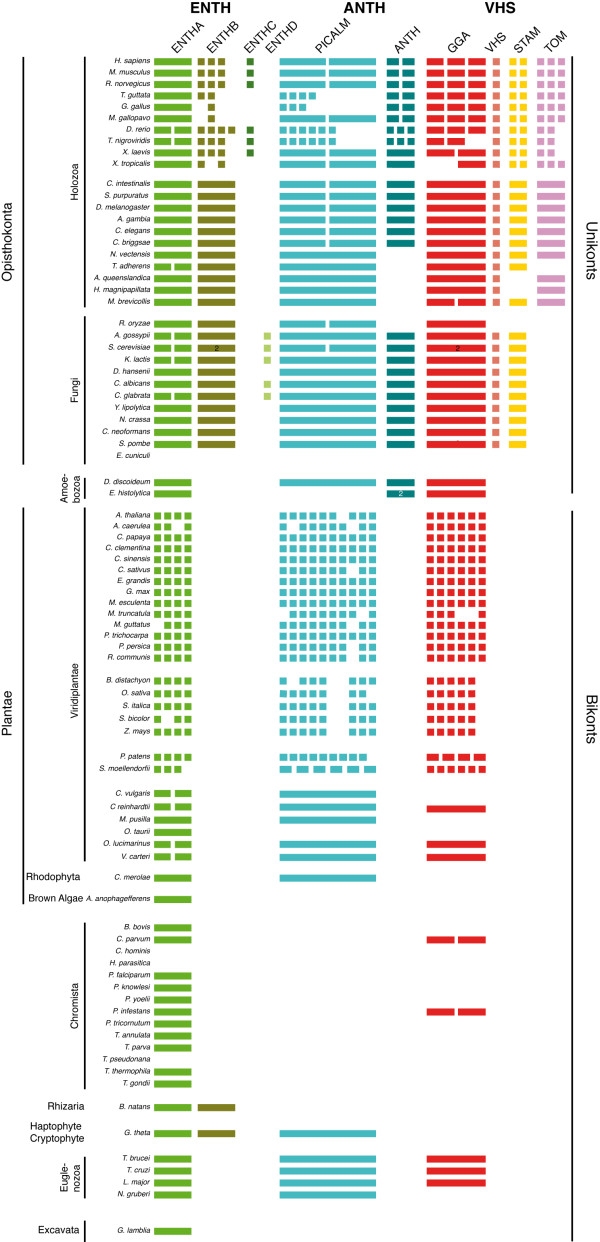
** Genomic distribution of the ENTH/ANTH/VHS superfamily members.** The presence or absence of all superfamily members was established by BLASTP searches. The number of rectangles in a subfamily of a given organism corresponds to the number of protein types in this subfamily. The number in a given type corresponds to the number of indistinguishable subtypes. Organism disposition is based on the phylogeny proposed by Cavalier-Smith (Cavalier-Smith 2010).

After validating our clustering, we assessed the genomic distribution of the ENTH/ANTH/VHS members in the analyzed species (Figure 2). The presence/absence profile recapitulates the proposed evolution of the endocytic system [17] with Metazoa and Fungi (Opisthokonta supergroup) being closer to the Amoebozoa than to Plantae, Chromista, Excavata, Rhizaria and Euglenozoa subfamilies. Opisthokonta possess the most complex panel with protein members in at least eight of the ten different subfamilies while Amoebozoa display four distinct subfamilies with the presence of ANTH-containing proteins (otherwise Opisthokonta-specific proteins) in the two studied organisms *D. discoideum* and *E. histolitica*. Plantae and Euglenozoa have representatives only in ENTHA, PICALM and GGA subfamilies while Chromista and Excavata only in the ENTHA subfamily (Figure 2). This suggests that the ENTHA, PICALM and GGA subfamilies conserved in different taxa were present prior to the formation of the different eukaryotic supergroups. 

The ENTHB subfamily (Human Epsin1-3 and yeast Ent1-2), which is required for endocytosis, is specific to Opisthokonta (Metazoa and Fungi) (Figure 2), suggesting that this subfamily was acquired more recently probably by duplication of the ENTHA subfamily. In addition, we identified two new Opisthokonta subfamilies of unknown function: ENTHC found in most vertebrates except Aves and ENTHD in the fungal subphylum Saccharomycotina (Figure 2).

Members of the ANTH family are also found in most taxa excluding the Chromista and Excavata, with the PICALM subfamily composed of Metazoa AP180 and CALM and Fungi yAP1801-2 being the most conserved. This is in agreement with a previous study showing that AP180 is an ancient component of the endocytic system [17]. In addition, our results show that the second ANTH subfamily (Metazoa HIP1(HSC70 Interacting Protein) and HIP1R, Fungi Sla2 and Amoeboza HIP1R), characterized by the I/LWEQ actin binding domain [27], is specific to Opisthokonta and Amoebozoa which form the Unikonts [28]. We do notice some exceptions with the Fungi *Rhizopus oryzae* and animalia *Monosiga brevicollis*, *Trichoplax adhaerens*, *Hydra magnapapillata*, *Nematostella vectensis* and *Amphimedon queenslandica* missing the ANTH proteins probably due to protein loss (Figure 2).

Analysis of the presence/absence profile in the VHS family shows that VHS and STAM subfamilies, forming the endosomal sorting complex ESCRT-0 are specific to Opisthokonta (Figure 2), as previously observed [18,23]. Here we also show that TOM proteins are specific to Metazoa and certainly a recent acquisition. Moreover in vertebrates, STAM and TOM proteins are duplicated or even triplicated, whereas the VHS subfamily contains only one member despite its crucial role in endosomal sorting [29]. This is even more surprising since this unique VHS protein directly interacts with the duplicated STAM proteins to form the ESCRT-0 complex [30]. Nonetheless, total absence of the ESCRT-0 complex is observed in the Fungi *Rhizopus oryzae* or partial in animalia *Hydra magnapapillata* and *Amphimedon queenslandica* which have a TOM but no STAM (Figure 2). *Trichoplax adhaerens* on the other hand has a STAM but no TOM protein. Thus, such organisms could be ideally suited to better characterize the function of these proteins.

Plantae, which undergo endocytosis and endosomal sorting [31,32], possess only ENTHA, PICALM and GGA subfamilies, all of which had a complex evolution as illustrated by the high number of duplications in each subfamily that we could group in several types (Figure 2). Despite being unable to assign clear functions to these different types, we could speculate on the potential functions of some ENTHA types. Indeed, Plantae ENTHA1, ENTHA2 types (*A. thaliana* Epsin1 and Epsin2/EpsinR2 respectively) and ENTHA3 cluster with *S. cerevisiae* Ent3 (yEnt3) and human EpsinR and could thus function in Golgi-to-endosomal sorting, while ENTHA4 type proteins may have a different role in trafficking, possibly endocytosis. Interestingly, the *A. thaliana* AtEpsin1 protein was shown to be required for Golgi sorting of vacuolar protein (similar functions as hEpsinR and yEnt3) and AtEpsinR2 binds to PtdIns3P suggesting an endosomal sorting function (similar as yEnt3) [33,34].

### Comparative genomic analysis

**Figure 3:**
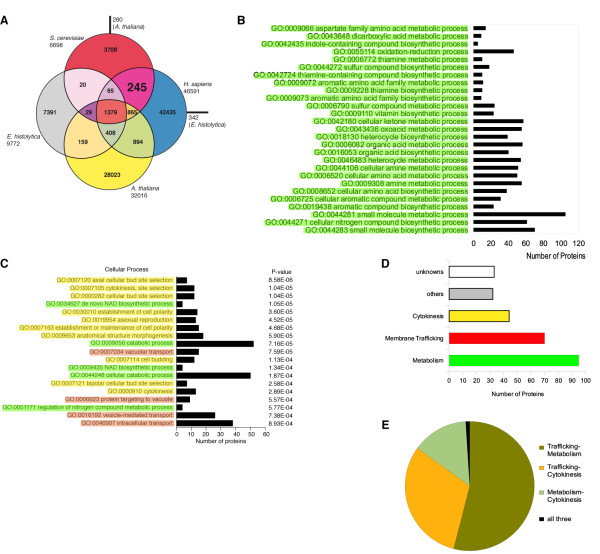
** Membrane trafficking is central for eukaryotic evolution.** (**A**) Distribution of proteins from *H. sapiens*, *S. cerevisiae*, *A. thaliana* and *E. histolytica* after reciprocal best hit analysis. Proteins of a given organism are assigned to one of the 15 categories according to the number of orthologues and the species in which they are found. (**B**) Analysis of the 245 proteins common to Opisthokonta and absent/divergent in Plantae and Protist by the *S. cerevisiae* GO (Gene Ontology) database. Only categories with a P-value inferior to 10^-4^ and comprising more than 3 proteins were retained. Categories highlighted in red are involved in cytokinesis, in yellow in membrane trafficking and in green in metabolic processes. (**C**) Analysis of the 280 proteins common to *S. cerevisiae* and *A. thaliana* by the *S. cerevisiae* GO database. The same category color code as used in (B) was applied. (**D**) The total number of proteins involved in each process (unknown function, other functions, trafficking, cytokinesis or metabolism) based on the *S. cerevisiae* GO database was represented and this irrespective of their involvement in more than one process. (**E**) Relative distribution of bifunctional proteins involved in trafficking-cytokinesis, trafficking-metabolism and cytokinesis-metabolism.

Membrane trafficking was proposed to be a key motor for the origin of eukaryotes via the acquisition of the endomembrane system [2]‐ [4]. Our genomic distribution of the members of the ENTH/ANTH/VHS superfamily results in a clustering of organisms similar to the tree of life (Figure 2), this suggests that trafficking was also a driving force in eukaryotic diversification. If indeed this hypothesis is true, then other trafficking proteins should display a similar distribution, i.e. present in Opisthokonta and absent in the other taxa. To test this, we performed a four way comparative genomic analysis on a database constituted of *H. sapiens*, *S. cerevisiae* (representatives of Opisthokonta), *A. thaliana* (Plantae) and *E. histolytica* (Amoebozoa) proteomes to identify the proteins that are specifically present in Opisthokonta and divergent/absent in Plantae and in Amoebozoa. Besides the *S. cerevisiae* proteome, we chose the proteomes of *H. sapiens* and *A. thaliana* because of their similar proteome size and the proteome of *E. histolytica* as a filter because it is the closest taxa to Opisthokonta and has been shown to be monophyletic hence it is divergent enough to obtain significant results (Figure 2) [17,35]. Furthermore its intracellular trafficking is fairly well characterized [36,37]. Each query protein was compared using a reciprocal best BLASTP hit to the database constituted of the four proteomes and classified in one of the 15 possible categories (Figure 3A). This led to the identification of 245 proteins common to *H. sapiens* and *S. cerevisiae* (Additional file 3: Figure S3)*,* 280 common to *A. thaliana* and *S*. *cerevisiae* and only 20 common to *S. cerevisiae* and *E. histolytica*. *A. thaliana* and *H. sapiens,* which have a similar proteome size (32016 and 46591 respectively), also have a similar number of proteins in common with *S. cerevisiae* (280 and 245 respectively), whereas baker’s yeast and amoeba (proteome size of 6698 and 9772 respectively) share only 20 proteins. The *S. cerevisiae* Gene Ontology (GO) annotation database was used to identify the biological processes in which these 245 proteins are involved, and the results were verified using the SGD (*Saccharomyces* Genome Database) database (Additional file 4: Figure S4). Among the 20 proteins common to *S. cerevisiae* and *E. histolytica* only one biological function, the chitin synthesis of the cell wall, was identified with a P-value below the 10^-4^ threshold and was not retained (Additional file 5 Figure S5). The different GO functions associated with proteins common to *A. thaliana* and *S*. *cerevisiae* fall into several categories all belonging to metabolism (Figure 3B and Additional file 5: Figure S5), whereas proteins common to *H. sapiens* and *S. cerevisiae* fall into three categories, membrane trafficking, cytokinesis and metabolism (Figure 3C and Additional file 4: Figure S4). The 17 GO categories involved in membrane trafficking, the 22 in cytokinesis and the 17 in metabolism contain 61, 37 and 81 different proteins respectively (Additional file 3: Figure S3). Among the 245 identified proteins, 102 are absent from our GO results of which 33 are of unknown function (in white, Additional file 3: Figure S3). For the remaining 69 proteins that did not form GO categories since less than 3 proteins shared the same GO term, we manually searched the SGD (*Saccharomyces* Genome Database) database and found that 46 proteins are involved in membrane trafficking, cytokinesis and/or metabolism and only 23 have other functions (Additional file 3: Figure S3). Thus, the overall analysis of the 245 proteins indicates that 73 are implicated in membrane trafficking, 97 in metabolism and 46 in cytokinesis (Figure 3C). Of the 73 proteins involved in membrane trafficking, 30 are involved in the endosomal network and 9 in secretion (based on the literature and on the SGD database). 

It is noteworthy that among the 245 proteins, 35 proteins share two of the three functions (i.e. intracellular trafficking and metabolism or cytokinesis)(Figure 3D and Additional file 3: Figure S3). Analysis of these 35 proteins shows that 20 proteins are involved in membrane trafficking and metabolism, 10 in trafficking and cytokinesis but only 4 in metabolism and cytokinesis and, 1 in the three processes (Figure 3D). These results further support the hypothesis according to which membrane trafficking was a key factor in eukaryotic evolution and probably influenced the evolution of the metabolic and cytokinesis functions.

## Discussion

All eukaryotes share a similar intracellular organization with the nucleus and membrane-bound organelles. Thus, they depend on similar requirements to transport newly synthesized proteins from the ER to their target organelle. Numerous phylogenetic studies have shown that many ubiquitous trafficking effectors, such as small GTPases, syntaxins, coat components or adaptors (such as COP or clathrin), the lipid PtdIns-kinases and -phosphatases involved in endocytosis and MVB sorting or the C2 domain found in many proteins involved in intracellular trafficking, the MABP (MVB12-associated β prism) domain present in ESCRT-I/MVB12 subunits and in other trafficking proteins and the UMA (UBAP1-MVB12-associated) domain found in regulators of ESCRT function, are highly conserved throughout the eukaryotic lineage [17,18,21,38]‐ [44]. The ENTH/ANTH/VHS superfamily of proteins is involved in three different trafficking pathways i.e. Golgi to endosomes, endosomal sorting and endocytic pathways forming the endosomal system. Here, we have revealed that although participating in the same pathways as the previously mentioned trafficking proteins, the different members and families of the ENTH/ANTH/VHS superfamily do not share the same evolutionary pattern.

Our results support the hypothesis that the ENTHA domain is the foundation of the superfamily since it is present in 80 out of the 84 studied eukaryotic organisms. In the Chromista and Excavata taxa, this domain is even the unique representative of the ENTH/ANTH/VHS function, thus suggesting that, in these organisms, multiple tasks might be performed by the unique ENTHA-containing protein. Accordingly, it is to note that, in *Trypanosoma brucei* (Euglenozoa), the ENTHA-containing protein localizes both as EpsinR (ENTHA) and Epsin (ENTHB) and is required for endocytosis [42]. In Plantae, we could define four different types of ENTHA (ENTHA1-4) (Figure 2). The *A. thaliana* Epsin1 (ENTHA1 in our analysis) and EpsinR2/Epsin2 (ENTHA2) proteins, were linked to functions in the Golgi to vacuole pathway [33,34]. The same could apply for ENTHA3, which is closely related to ENTHA1 and ENTHA2 in our analysis. We also identified another ENTHA member, the ENTHA4 that clusters on another branch (Figure 1) and may represent the epsin required for endocytosis. All these evidences support our hypothesis of a multi-functional role in trafficking for this ENTHA subfamily in organisms lacking the ENTHB subfamily. The Plantae GGA and PICALM domains diverged early on in at least two branches (giving rise to different types, Figure 1 and 2). The *A. thaliana* AP180 (At1g05020, NP_563726) protein (Picalm6 in our analysis) was shown to be involved in clathrin-mediated endocytosis and specifically recruited at the plasma membrane upon PdtIns(4,5)*P*_2_ production [45,46]. Based on the phylogenetic tree (Figure 1) and on the MACS of the full length proteins, this Picalm6 could be functionally redundant in endocytosis with the Picalm4-5 proteins (At4g32285, At2g25430; At4g02650, At1g03050), whereas the Picalm9-10 proteins (At1g68110, At1g25240, At1g14686, At2g01920; At4g40080, At5g10410, At5g65370) that are clustered on a separated branch of the phylogenetic tree might be required for other plant endocytic functions [47]. Here, we also show that the Plantae, Euglenozoa and Amoebozoa VHS proteins belong to the GGA subfamily, even though they lack the GAE C-terminal domain, what led to their classification in the TOM subfamily [21]‐ [23]. The biological and trafficking function of the TOM proteins are very different from the ones displayed by the GGA proteins. Indeed, the GGA proteins are required for protein sorting at the trans Golgi network, via direct binding between their VHS domain and sorting motifs present on the cargos [48,49]. Since Plantae GGA proteins are clustered in different types, we propose that some perform Opisthokonta GGA functions, while others mimic the VHS and STAM functions that are required for cargo sorting at the endosomes in conjunction with the ESCRT-I complex and the Vps4 ATPase [18,50]. The Euglenozoa and Amoebozoa GGA protein could be multifunctional for cargo sorting at the Golgi and at the endosomes, since in *D. discoidium* it interacts with DdTsg101 an endosomal ESCRT-I subunit and with clathrin that is required for Golgi trafficking [22]. In conclusion, the large duplication of these three ENTHA, PICALM and GGA subfamilies in Plantae might allow the different subtypes to function at different steps of trafficking, and thus did not require the emergence of other subfamilies. Therefore, this study should advance the understanding of the ENTH/ANTH/VHS superfamily proteins in Plantae and in protists.

Our analysis of a large number of fully sequenced genomes representative of the different eukaryotic taxa allowed us to observe a strong correlation between the presence/absence/duplication of members of this superfamily and the eukaryotic evolutionary tree (Figure 2). Indeed, most of the different eukaryotic kingdoms (Opisthokonta, Amoebozoa, Plantae, Euglenozoa) can easily be distinguished based solely on the number of subfamilies populated by at least one protein (Figure 2). Only the Excavata and Chromista kingdoms could not be distinguished from each other having only the ENTHA protein (Figure 2). Moreover, among the Opisthokonta, Animalia and Fungi kingdoms can be clustered in two separate groups, based on the presence/absence of the TOM subfamily. The TOM subfamily as well as the VHS and STAM subfamily probably results from the duplication and divergence of the GGA subfamily to fulfill the needs of a more complex organelle organization. Several *in vivo* studies on different model organisms have shown that in Opisthokonta vesicular budding and cargo sorting at the different steps of the endosomal system requires the combination of two proteins from different ENTH/ANTH/VHS subfamilies [51]‐ [53]). This implies that these partners had to coevolve. This hypothesis is supported by our results showing that the absence of one partner results also in the lack of the other protein, such as the pair ENTHB/ANTH or VHS/STAM (S.c. Ent1/Sla2 or H.s. Hrs/STAM). For most Plantae proteins identified in this analysis the biological function is unknown, however a similar pairing rule could apply between the protein types.

Our genomic distribution also suggests that membrane trafficking was a driving force for the eukaryotic diversification in different taxa. This hypothesis was tested by comparative genomics and GO enrichment analyses and resulted in the identification of membrane trafficking, metabolism and cytokinesis as the only cellular functions differentiating Opisthokonta (*H. sapiens* and *S. cerevisiae*) from Plantae (*A. thaliana*) and Amoebozoa (*E. histolytica*), only metabolic functions were common to *S. cerevisiae* and *A. thaliana*, and none could statistically be considered common to *S. cerevisiae* and *E. histolytica*. Among the 75 proteins identified as Opisthokonta specific and involved in trafficking, 30 were involved in the endosomal system supporting our hypothesis that this process contributed to the evolution of Opisthokonta. It is also noteworthy that of these same 75 proteins, 20 have overlapping functions in intracellular trafficking and metabolism and 10 in intracellular trafficking and cytokinesis, a specificity of Opisthokonta. In both cases, the majority of proteins are involved in endosomal network. These results further support the endosomal system as an evolutionary driving force hypothesis.

## Conclusions

In conclusion, although intracellular trafficking connects the same organelles in all eukaryotes and involves common regulators such as Rab GTPases, SNAREs or Clathrin, the ENTH/ANTH/VHS superfamily shows a distinct distribution between Opisthokonta and Plantae [17]. Furthermore, without being able to assign a function to proteins with a TOM domain we could definitely conclude that they are a Metazoa specific subfamily of proteins and that ENTHA and GGA (involved in Golgi to endosome transport) and PICALM (involved in endocytosis) were present in eukaryotes prior to their evolution in the different taxa. Finally, our results show that membrane trafficking was tightly linked to cytokinesis and metabolism in Opisthokonta, this strongly supports a central role of membrane trafficking in the formation of the Opisthokonta and Amoebozoa taxa. This also suggests that membrane trafficking was not only a key acquisition for eukaryogenesis, but was also crucial for the emergence of different eukaryotic taxa, probably by allowing complex innovative metabolic pathways to be organized in compartments to protect cell homeostasis.

## Methods

### Generation of the multiple alignment of complete sequences

Amino acid sequences of proteins with an ENTH, ANTH or VHS domain were gathered for 20 Metazoa (*Homo sapiens, Mus musculus, Rattus norvegicus, Gallus gallus, Meleagris gallopavo, Taeniopygia guttata, Tetraodon nigroviridis, Denio rerio, Xenopus laevis, Xenopus tropicalis, Drosophila melanogaster, Anopheles gambia, Caenorhabditis elegans, Caenorhabditis briggsae, Strongylocentrotus purpuratus, Nematostella vectensis,Trichoplax adhaerens, Monosiga brevicollis*, *Hydra magnipapillata* and *Ciona intestinalis*), 12 Fungi (*Saccharomyces cerevisiae, Ashbya gossypii, Schizosaccharomyces pombe, Candida albicans, Candida glabrata, Kluyveromyces lactis, Debaryomyces hansenii, Yarrowia lipolytica, Neurospora crassa, Rhizopus oryzae, Cryptococcus neoformans and Encephalitozoon cuniculi)*, 21 plants (*Arabidopsis thaliana, Populus trichocarpa, Aquilegiacoerulea*, *Carica papaya*, *Citrus clementina*, *Citrus sinesis*, *Cucumis sativa*, *Eucalyptus grandis*, *Glycina max*, *Manihot esculenta*, *Medicago truncatula*, *Mimulus guttatus*, *Prunus persica*, *Ricinus communis, Brachypodium distachyon,Setaria italica*, *Sorghum bicolor*, *Zea mays*, *Physcomitrella patens*, *Selaginella moellendorfii* and *Oryza sativa*), 1 brown alga ( *Aureoccocus anophagefferens)*, 1 red alga *(Cyanidioschyzon merolae*), 6 green algae *(Micromonas pusilla, Ostreoccocus taurii, Ostreoccocus lucimarinus, Chlamydomonas reinhardtii, Volvox carteri* and *Chlorella vulgaris*) and 23 protists (*Babesia bovis*, *Guillardia theta, Hyaloperonospora parasitica*, *Phytophtora infestans*, *Plasmodium knowlesi, Phaeodactylum tricornutum, Theileria annulata*, *Toxoplasma gondii*, *Naegleria gruberi*, *Bigelowiella natans*, *Trypanosoma brucei, Trypanosoma cruzi, Leishmania major, Dictyostelium discoideum, Entamoeba histolytica, Tetrahymena thermophila, Giardia lamblia*, *Plasmodium falciparum*, *Plasmodium yoelii*, *Theileria parva*, *Cryptosporidium parvum*, *Thalassiosira pseudonana* and *Cryptosporidium hominis*) from the NCBI, Ensembl and the Joint Genome Institute (JGI) databanks. Sequences were blasted (using BLASTP, TBLASTN and PSI-BLAST to ascertain the absence of any missing protein) and aligned using PipeAlign cascade [54] and were manually adjusted.

The ∝ − helices of the ENTH, ANTH and VHS domains were localized on the Multiple Alignment of Complete Sequences (MACS) based on the protein structures found in the PDB database. Structure of TOM is 1ELK [24], VHS is 1DVP [8], Epsin1 is 1INZ [55], GGA1 is 1JWF [25], CALM is 1HF8 [9] and STAM is 1X5B (by Tochio, N., Koshiba, S., Inoue, M., Kigawa, T. and Yokoyama, S. for the RIKEN Structural Genomics/Proteomics Initiative (RSGI)).

To identify the domains and motifs present in the proteins, the MACS was scanned using Interproscan and the InterPro database [56].

### Phylogeny

In order to reconstruct the phylogeny, we used the ∝ − helices 2 to 7 of the VHS, ENTH and ANTH domains. We excluded the following sequences because of incompleteness or poor prediction: *R. norvegicus* Tom1 np_001011994, *O. Sativa* np_001062546, *X. tropicalis* Tom1 np_001079451 and *T. nigroviridis* ANTH2. We used the neighbor joining algorithm implemented in Phylowin with 500 bootstraps to generate the phylogenetic tree [57]. For tree visualization and editing we used iTOL [58].

### Reciprocal best hit analysis

We used the proteomes of *S. cerevisiae* from http://www.yeastgenome.org/, *H. sapiens* from http://www.ncbi.nlm.nih.gov, *A. thaliana* from http://www.plantgdb.org/AtGDB/ and *E. histolytica* from the http://amoebadb.org to generate our database. Each of the 95 077 proteins was compared to the whole database using BLASTP [59] with a cutoff E value of 10^-10^. This allowed the classification of the proteins in 15 groups according to the number of organisms in which each protein had an ortholog.

Proteins from the yeast-human group were then manually curated to eliminate proteins with coverage lower than 20% by BLASTP. The remaining 245 proteins were analyzed by AmiGO Term Enrichment on the Gene Ontology website using the *Saccharomyces cerevisiae* database. The cutoff for the p-value was set to 10^-4^. The GO category was retained only if it comprises more than 3 proteins among the 245 proteins analyzed. We also manually searched the SGD (*Saccharomyces* Genome Database) database to verify their proper assignment into a given GO category and to complete the functional analysis for the proteins that did not form GO categories since less than 3 proteins shared the same GO term. The yeast-tale cress (280 proteins) and yeast-amoeba (20 proteins) groups, not manually curated, were also analyzed by AmiGO Term Enrichment on the Gene Ontology website using the *Saccharomyces cerevisiae* database.

## Competing interests

The authors declare that they have no competing interests.

## Authors’ contributions

JODC, SF, JT and OP conceived, designed and guided the study. JODC, SF, OP and JT drafted the manuscript, OL helped to draft the manuscript. JODC performed the bioinformatics analysis. RR, performed the reciprocal best hit analysis. JODC performed the sequence alignment with the help of OL, JT and OP. JODC, SF, OL, JT, and OP analysed the data. All authors read and approved the final manuscript.

## Supplementary Material

Additional file 1 ** Figure S1.** Phylogeny of the proteins with a GAT domain. The sequence of most proteins with a GAT domain were analyzed, excluding sequences CAF91904 and CAF95287 from *Tetraodon nigroviridis*, NP_973770 and NP_850834 from *Arabidopsis thaliana* and EDM05672 from *Rattus norvegicus* due to bad predictions. The phylogenetic tree was calculated using SeaView by parsimony with 500 bootstrapped replications [57]. The tree display was performed by iTOL and the tree re-rooted at the base of the TOM branch [58]. (*) denotes wrongly attributed taxa for NP_00112339 (TOMl1 *X. tropicalis*) in GGA and NP_572571 (GGA *D. melanogaster*) in Fungi GGA. Click here for file

Additional file 2 ** Figure S2.** Aligment of the VHS domains of GGA and TOM proteins. (A) The sequences of the VHS domain of Metazoa, Fungi, Plantae and Prostists GGA were aligned and the secondary structure of human GGA1 is shown above the sequence alignment (PDB ID code 1JWF). (B) The sequences of the VHS domain of Metazoa TOM were aligned and the secondary structure of human TOM1 is shown above the sequence alignment (PDB ID code 1ELK). Arrows indicate features specific to the TOM family. ESPript website was used for displaying purposes [60]. Click here for file

Additional file 3 ** Figure S3.** List of Opisthokonta specific proteins identified by the 4 way comparative genomic analysis. Among the 320 proteins specific to *H.* s*apiens* and *S.* c*erevisiae* that were found by comparative genomic analysis, 245 proteins were confirmed and kept for further GO Term Enrichment analysis and 75 proteins were rejected. To identify the biological processes in which these 245 confirmed proteins were involved, we used the *S. cerevisiae* Gene Ontology (GO) annotation database and we also manually searched the SGD (*Saccharomyces* Genome Database) database. We highlighted the proteins involved in membrane trafficking (in red), in cytokinesis (in yellow), in metabolism (in chartreuse green), in trafficking and cytokinesis (in orange), in trafficking and metabolism (in khaki green), in metabolism and cytokinesis (in light green) and in the three processes in black. The proteins that did not belong to any of these catagories were highlighted in grey for the proteins involved in other biological processes and not highlighted proteins are of unknown function. Click here for file

Additional file 4 ** Figure S4.** GO Term Enrichment of Opisthokonta proteins. The complete analysis of the yeast *S. cerevisiae* GO Term Enrichment analysis for cellular processes is shown. The categories with a P-value inferior to 10^-3^ and comprising more than 3 proteins were retained. Biological processes involved in metabolism are highlighted in green, trafficking in red and cytokinesis in yellow. Click here for file

Additional file 5 ** Figure S5.** GO Term Enrichment of yeast-plant and yeast-protist proteins.The complete analysis of the yeast *S. cerevisiae* GO Term Enrichment analysis for cellular processes is shown, only categories with a P-value inferior to 10^-3^ and comprising more than 3 proteins were retained. (A) Proteins common to *S. cerevisiae* and *A. thaliana* are all involved in metabolism (mainly in amino-acids and some vitamins biosynthesis pathways) and highlighted in green. (B) Proteins common to *S*. *cerevisiae* and *E. histolytica* are involved in the chitin metabolism. Chitin is a major component of the yeast cell wall and of the Entamoeba cyst wall. (PDF 426 kb) Click here for file
