# Supplementary figures and images for: Evolutionary analysis of the ENTH/ANTH/VHS protein superfamily reveals a coevolution between membrane trafficking and metabolism
Source: BMC Genomics. 2012 Jul 2;13:297. doi: 10.1186/1471-2164-13-297 (PMC3473312; doi:10.1186/1471-2164-13-297)

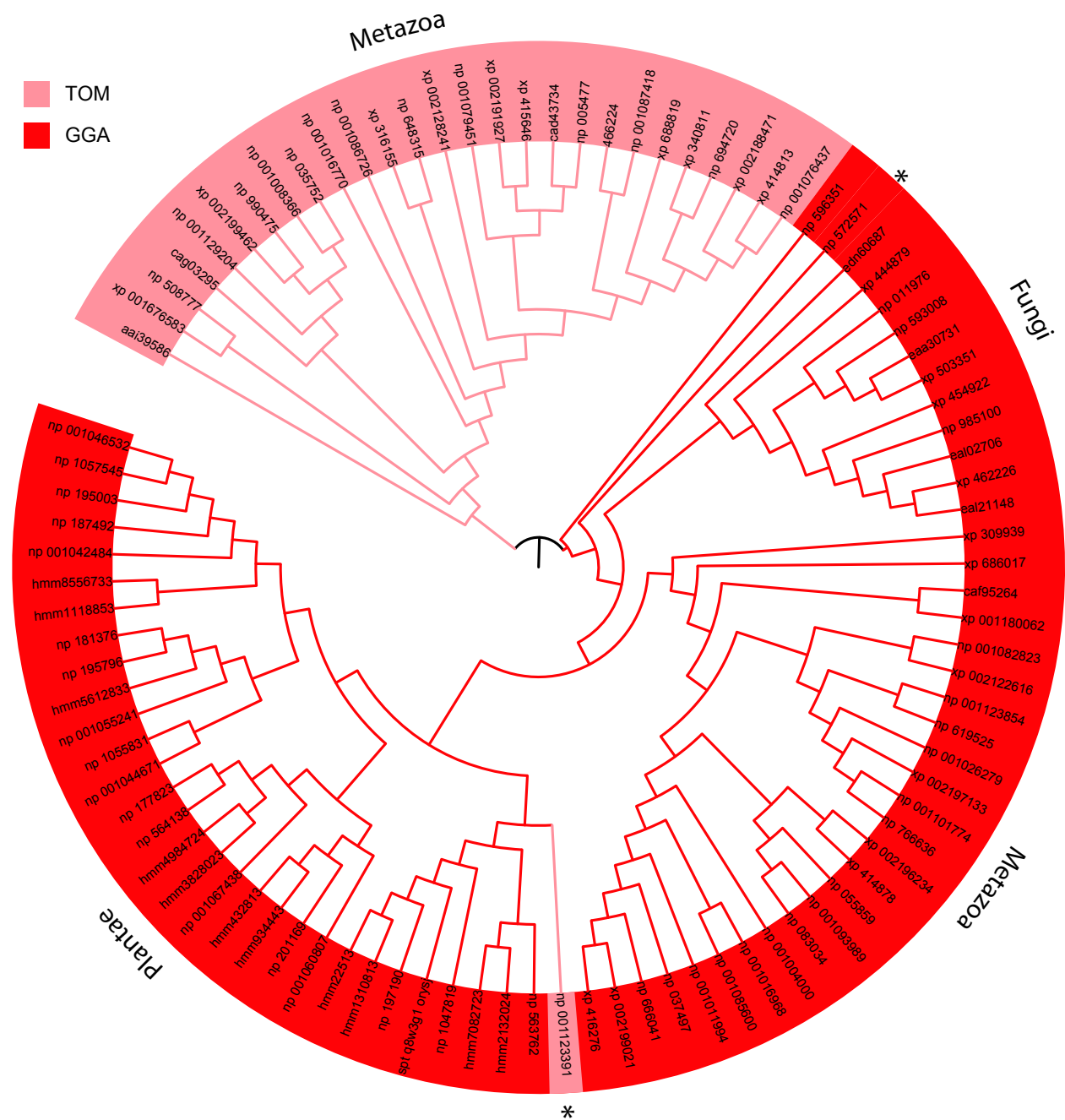

Supplement: Additional file 1 — Figure S1. Phylogeny of the proteins with a GAT domain. The sequence of most proteins with a GAT domain were analyzed, excluding sequences CAF91904 and CAF95287 from Tetraodon nigroviridis, NP_973770 and NP_850834 from Arabidopsis thaliana and EDM05672 from Rattus norvegicus due to bad predictions. The phylogenetic tree was calculated using SeaView by parsimony with 500 bootstrapped replications [57]. The tree display was performed by iTOL and the tree re-rooted at the base of the TOM branch [58]. (*) denotes wrongly attributed taxa for NP_00112339 (TOMl1 X. tropicalis) in GGA and NP_572571 (GGA D. melanogaster) in Fungi GGA. [file 1471-2164-13-297-S1.pdf]
