# Supplementary material for: Evolutionary analysis of the ENTH/ANTH/VHS protein superfamily reveals a coevolution between membrane trafficking and metabolism
Source: BMC Genomics. 2012 Jul 2;13:297. doi: 10.1186/1471-2164-13-297 (PMC3473312; doi:10.1186/1471-2164-13-297)
Supplement: Additional file 3 — Figure S3. List of Opisthokonta specific proteins identified by the 4 way comparative genomic analysis. Among the 320 proteins specific to H. sapiens and S. cerevisiae that were found by comparative genomic analysis, 245 proteins were confirmed and kept for further GO Term Enrichment analysis and 75 proteins were rejected. To identify the biological processes in which these 245 confirmed proteins were involved, we used the S. cerevisiae Gene Ontology (GO) annotation database and we also manually searched the SGD (Saccharomyces Genome Database) database. We highlighted the proteins involved in membrane trafficking (in red), in cytokinesis (in yellow), in metabolism (in chartreuse green), in trafficking and cytokinesis (in orange), in trafficking and metabolism (in khaki green), in metabolism and cytokinesis (in light green) and in the three processes in black. The proteins that did not belong to any of these catagories were highlighted in grey for the proteins involved in other biological processes and not highlighted proteins are of unknown function. [file 1471-2164-13-297-S3.pdf]

Proteins	Rejected Yeast	Rejected Human	Kept Yeast	Kept Human
1	Ade8	GART	Ace2	KLF16
2	Akr2	ZDHHC17	Ade13	ADSL
3	Bcd1	C1orf181	Aim10	PARS2
4	Bdp1	BTF3	Aim17	TMLHE
5	Btt1	BTF3	Aim22	LIPT1
6	Cbp3	C20orf44	Aim29	C2orf76
7	Cox19	COX19	Air2	ZCCHC7
8	Cup9	PKNOX2	Ams1	MAN2C1
9	Ecm5	JARID1C	Apn2	APEX2
10	Erg28	C14orf1	Arg82	IHPK1
11	Erp1	TMED9	Aro8	AADAT
12	Erp6	TMED9	Aro9	AADAT
13	Far10	SLMAP	Atg2	C14orf103
14	Fmt1	MTFMT	Ath1	ATHL1
15	Fps1	AQP9	Atp11	ATPAF1
16	Gim4	PFDN2	Avl9	KIAA0241
17	Gtb1	PRKCSH	Bar1	PGC
18	Ist1	AC009127	Bet5	TRAPPC1
19	Kap114	IPO9	Bna1	HAAO
20	Lsb6	PI4K2B	Bna2	INDOL1
21	Maf1	MAF1	Bna4	KMO
22	Mkc7	PGC	Bna5	KYNU
23	Mog1	RANGRF	Bro1	PDCD6IP
24	Ngl3	PDE12	Bts1	GGPS1
25	Nic96	NUP93	Bub1	BUB1
26	Nip100	AL163953	Bud20	ZNF593
27	Npl4	NPLOC4	Bzz1	FNBP1L
28	Nup116	NUP98	Cat2	CRAT
29	Nup2	RANBP2	Cat5	COQ7
30	Pac1	PAFAH1B1	Cch1	NALCN
31	Pac2	TBCE	Cdc10	40057
32	Pam18	DNAJC	Cdc11	38596
33	Pho81	ANK2	Cdc12	39326
34	Pol4	POLL	Cdc14	CDC14A
35	Pop4	POP4	Cdc3	39326
36	Por1	VDAC3	Cdd1	CDA
37	Por2	VDCA1	Cgi121	AC074008
38	Psf1	RP4-691N24	Csn12	PCID2
39	Ptk1	TSSK6	Cha1	SDSL
40	Ptp2	PTPRC	Cox6	COX5A
41	Rce1	RCE1	Cps1	PM20D1
42	Rna1	RANGAP1	Csf1	KIAA1109
43	Rpb4	POLR2D	Cwh43	AC020593
44	Rpn4	PAAF1	Cyc3	HCCS
45	Rpp1	RP11-320F15	Cyt2	HCCS
46	Rps28a	RPS28	Dan4	C14orf1
47	Rps28b	RPS28	Dal2	ALLC
48	Rrp1	KIAA179	Dci1	AC007562
49	Rtt103	C20orf77	Dot1	DOT1L
50	Sap185	SAPS1	Dug1	CNDP2
51	Ser2	PSPH	Dug2	CNDP1
52	Sfl1	HSF4	Dyn1	DYNC1H1
53	Shy1	SURF1	Eci1	CDYL
54	Sip2	PRKAB2	Ecm14	CPA4
55	Ski3	KIAA0372	Egd2	NACA
56	Snf4	PRKAG2	Elm1	CAMKK1
57	Snf5	SMARCB1	Emc4	TMEM85
58	Srp40	NOLC1	Emi5	C11orf79
59	Tan1	THUMPD1	Ena2	TMED9
60	Tfb1	GTF2H1	End3	EPS15
61	Tfc4	GTF3C3	Ent4	EPN1
62	Thi80	TPK1	Epl1	EPC2
63	Trs23	TRAPPC4	Erg2	OPRS1
64	Ydl114w	DHRS8	Erg27	HSD17B7
65	Ydr333c	TCF25	Ero1	MTFMT
66	Yer066w	FBXW7	Erp4	TMED5
67	Yer152c	AADAT	Erv29	SURF4
68	Ygr021w	CCDC45	Far11	FAM40A
69	Ygr109w-b	RTL1	Fat1	SLC27A2
70	Yhi9	PBLD	Fau1	MTHFS
71	Yhm2	SLC25A21	Fip1	FIP1L1
72	Yil082w-a	RTL1	Fkh1	FOXJ1
73	Ylr072w	GRAMD1A	Fkh2	FOXK2
74	Ymd8	SLC35C2	Fmp30	NAPEPLD
75	Ymr178w	FLAD1	Fmp42	SLC43A3
76	Ymr291w	DCLK3	Fsf1	SFXN5
77	Ynl181w	RDH14	Fzf1	KLF11
78	Yps1	PGA5	Gal7	GATLT
79	Zim17	C9orf151	Gat1	GATA4
80			Get4	C7orf20
81			Gga2	GGA3
82			Gip2	PPP1R3C
83			Glg2	GYG2
84			Grh1	GORASP1
85			Gsh1	GCLC
86			Gsy1	GYS1
87			Gsy2	GSY1
88			Gzf3	GATA6
89			Hcm1	FOXL2
90			Hem14	PPOX
91			Hgh1	C8orf30B
92			Hse1	STAM2
93			Iml1	DEPDC5
94			Iqg1	IQGAP3
95			Isc1	SMPD2
96			Isd11	LYRM4
97			Jac1	HSCB
98			Jhd1	FBXL11
99			Kcs1	IHPK3
100			Kex2	PCSK7
101			Laa1	HEATR5A
102			Lap3	BLMH
103			Lrg1	ARHHAP6
104			Lsb1	SH3D19
105			Lsm12	LSM12
106			Mch3	SLC16A12
107			Mch4	SLC16A3
108			Mch5	SLC16A12
109			Mcm1	SRF
110			Meu1	MTAP
111			Mia40	CHCHD4
112			Mip1	POLG
113			Mrm2	FTSJ2
114			Mrps5	MRPS5
115			Msl1	SNRPA
116			Mup1	SLC7A9
117			Mvp1	SNX8
118			Nas2	PSMD9
119			Nop13	HNRPD
120			Npr2	TUSC4
121			Nte1	PNPLA7
122			Nuc1	ENDOG
123			Nup157	NUP155

Proteins	Kept Yeast	Kept Human
124	Nup84	NUP107
125	Nus1	NUS1
126	Oar1	POLL
127	Opi3	PEMT
128	Orc4	ORC4L
129	Ost3	MAGT1
130	Pan3	PAN3
131	Pbp2	PCBP3
132	Pcp1	PARL
133	Pdc2	TIGD4
134	Pde2	PDE4B
135	Pef1	PDCD6
136	Pep1	SORCS2
137	Pep12	STX12
138	Pex13	PEX13
139	Pgs1	PGS1
140	Pho8	ALPPL2
141	Pig2	PPP1R3C
142	Pin3	GRB2
143	Pmt2	POMT2
144	Pmt3	POMT2
145	Pmt4	POMT2
146	Pmt6	POMT2
147	Pnp1	NP
148	Ppx1	PRUNE
149	Prb1	PCSK9
150	Pxl1	LHX8
151	Pxr1	AC105001
152	Qcr7	UQCRB
153	Rad14	XPA
154	Rga1	ARHGAP23
155	Rmd1	RMND1
156	Rmd8	RMND1
157	Rpc34	POLR3F
158	Rpl22a	RPL22L1
159	Rpl22b	RPL22L1
160	Rrp42	EXOSC7
161	Rrp46	EXOSC5
162	Rrt12	PCSK9
163	Rsc1	PBRM1
164	Rsc2	PBRM1
165	Rtc1	WDR24
166	Rvs161	BIN3
167	Sam50	SAMM50
168	Sap190	SAPS1
169	Scp1	TAGLN3
170	Scp160	HDLBP
171	Sdp1	DUSP14
172	Sec15	EXOC6
173	Sec62	SEC62
174	Sen34	TSEN34
175	Set7	SETD6
176	Shs1	39698
177	Smp3	PIGZ
178	Snt1	NCOR1
179	Snx3	SNX3
180	Sok1	TCP11L2
181	Spr28	39326
182	Spr3	39326
183	Spt3	SUPT3H
184	Sro7	STXBP5L
185	Srx1	SRXN1
186	Sto1	NCBP1
187	Sub1	SUB1
188	Svp26	TEX261
189	Swc5	DHRS7B
190	Swi5	KLF1
191	Tdp1	TDP1
192	Tes1	ACOT8
193	Tfb3	MNAT1
194	Thr4	THNSL2
195	Tid3	KNTC2
196	Tim13	TIMM13
197	Toa1	GTF2A1
198	Tom70	TOMM70A
199	Tpa1	OGFOD1
200	Trm44	C4orf23
201	Tsc10	FVT1
202	Tsc11	AC026713
203	Twf1	AC097637
204	Ubr1	UBR2
205	Ubx3	FAF2
206	Ups1	PRELID1
207	Vam6	VPS39
208	Vid24	C17orf39
209	Vid30	RANBP10
210	Vps1	FLAD1
211	Vps27	HRS
212	vps60	CHMP5
213	Vps74	GOLPH3L
214	Vps8	VPS8
215	Vth1	SORT1
216	Vth2	SORT1
217	Yat1	CPT2
218	Yat2	CPT2
219	Ybl104c	AC004982
220	Ybr220c	SLC33A1
221	Ydl114w	DHRS8
222	Ydl177c	IMPACT
223	Ydr282c	RMND1
224	Ydr307w	POMT2
225	Yea4	SLC35B4
226	Yer134c	MDP-1
227	Yfr018c	QPCT
228	Ygr015c	ABHD11
229	Ygr031w	ABHD11
230	Yhr033w	ALDH18A1
231	Yim1	RTN4IP1
232	Ylr422w	DOCK3
233	Ymr027w	RP11-108N8
234	Ymr034c	TIGD3
235	Ymr185w	TMCO7
236	Ymr253c	TMEM20
237	Ymr259c	THADA
238	Ynl040w	AARSD1
239	Ynr065c	SORL1
240	Yol114c	ICT1
241	Yor093c	DIP2B
242	Ypl162c	MUSTN1
243	Ypl225w	Cxorf26
244	Ypl264c	TMEM20
245	Ysp3	PCSK9

metabolism
trafficking
cytokinesis
trafficking+ metabolism
trafficking+ cytokinesis
metabolism+ cytokinesis
all three
others
unknown
