# Supplementary material for: Evolutionary analysis of the ENTH/ANTH/VHS protein superfamily reveals a coevolution between membrane trafficking and metabolism
Source: BMC Genomics. 2012 Jul 2;13:297. doi: 10.1186/1471-2164-13-297 (PMC3473312; doi:10.1186/1471-2164-13-297)
Supplement: Additional file 4 — Figure S4. GO Term Enrichment of Opisthokonta proteins. The complete analysis of the yeast S. cerevisiae GO Term Enrichment analysis for cellular processes is shown. The categories with a P-value inferior to 10-3 and comprising more than 3 proteins were retained. Biological processes involved in metabolism are highlighted in green, trafficking in red and cytokinesis in yellow. [file 1471-2164-13-297-S4.pdf]

Biological processes between S. cerevisiae and Human

GO Term	P-value	Sample frequency	Background frequency	Genes
GO:0007120 axial cellular bud site selection	8,58E-006	7/245 (2.9%)	21/6346 (0.3%)	ELM1 CDC3 CDC10 SHS1 CDC11 CDC12 PEF1
GO:0007105 cytokinesis, site selection	1,04E-005	12/245 (4.9%)	69/6346 (1.1%)	END3 ELM1 CDC3 CDC10 BUD20 SHS1 RVS161 SEC15 CDC11 CDC12 TWF1 PEF1
GO:0000282 cellular bud site selection	1,04E-005	12/245 (4.9%)	69/6346 (1.1%)	END3 ELM1 CDC3 CDC10 BUD20 SHS1 RVS161 SEC15 CDC11 CDC12 TWF1 PEF1
GO:0034627 de novo NAD biosynthetic process	1,05E-005	4/245 (1.6%)	5/6346 (0.1%)	BNA1 BNA5 BNA4 BNA2
GO:0034354 de novo NAD biosynthetic process from tryptophan	1,05E-005	4/245 (1.6%)	5/6346 (0.1%)	BNA1 BNA5 BNA4 BNA2
GO:0030010 establishment of cell polarity	3,60E-005	14/245 (5.7%)	103/6346 (1.6%)	END3 ELM1 CDC3 CDC10 BUD20 SHS1 RVS161 SRO7 SEC15 CDC11 CDC12 TWF1 RGA1 PEF1
GO:0019954 asexual reproduction	4,52E-005	13/245 (5.3%)	92/6346 (1.4%)	END3 ELM1 CDC3 CDC10 SHS1 RVS161 SEC15 CDC11 CDC12 TWF1 RGA1 LRG1 PEF1
GO:0007163 establishment or maintenance of cell polarity	4,68E-005	15/245 (6.1%)	119/6346 (1.9%)	PXL1 END3 ELM1 CDC3 CDC10 BUD20 SHS1 RVS161 SRO7 SEC15 CDC11 CDC12 TWF1 RGA1 PEF1
GO:0009653 anatomical structure morphogenesis	5,90E-005	18/245 (7.3%)	165/6346 (2.6%)	SPR3 PXL1 END3 ELM1 CDC3 CDC10 BUD20 SHS1 RVS161 SRO7 SEC15 CDC11 CDC12 TWF1 SPR28 RGA1 LRG1 PEF1
GO:0000902 cell morphogenesis	5,90E-005	18/245 (7.3%)	165/6346 (2.6%)	SPR3 PXL1 END3 ELM1 CDC3 CDC10 BUD20 SHS1 RVS161 SRO7 SEC15 CDC11 CDC12 TWF1 SPR28 RGA1 LRG1 PEF1
GO:0032989 cellular structure morphogenesis	5,90E-005	18/245 (7.3%)	165/6346 (2.6%)	SPR3 PXL1 END3 ELM1 CDC3 CDC10 BUD20 SHS1 RVS161 SRO7 SEC15 CDC11 CDC12 TWF1 SPR28 RGA1 LRG1 PEF1
GO:0048856 anatomical structure development	5,90E-005	18/245 (7.3%)	165/6346 (2.6%)	SPR3 PXL1 END3 ELM1 CDC3 CDC10 BUD20 SHS1 RVS161 SRO7 SEC15 CDC11 CDC12 TWF1 SPR28 RGA1 LRG1 PEF1
GO:0009056 catabolic process	7,16E-005	52/245 (21.2%)	797/6346 (12.6%)	BNA1 BRO1 GAL7 ISC1 CHA1 LAP3 VID24 TFB3 GGA2 CPS1 GAT1 ATH1 AMS1 DUG1 CDD1 ATG2 PEP1 STO1 PHO8 ECI1 RRP46 RRP42 NPR2 TPA1 PNP1 PPX1 DCI1 APN2 DAL2 CSF1 GSH1 AIR2 YSP3 NTE1 HEM14 JHD1 ARG82 NUC1 VAM6 VID30 ARO9 NAS2 DUG2 YEA4 PRB1 DAN4 BAR1 YNR065C YMR034C VPS8 RAD14 PEF1
GO:0007034 vacuolar transport	7,59E-005	15/245 (6.1%)	124/6346 (2.0%)	BRO1 VID24 VPS27 VPS60 GGA2 MVP1 ATG2 PEP1 VTH1 VPS1 VAM6 VTH2 VPS8 HSE1 PEP12
GO:0007114 cell budding	1,13E-004	12/245 (4.9%)	87/6346 (1.4%)	END3 ELM1 CDC3 CDC10 SHS1 RVS161 SEC15 CDC11 CDC12 TWF1 RGA1 PEF1
GO:0009435 NAD biosynthetic process	1,34E-004	4/245 (1.6%)	8/6346 (0.1%)	BNA1 BNA5 BNA4 BNA2
GO:0032506 cytokinetic process	1,75E-004	12/245 (4.9%)	91/6346 (1.4%)	END3 ELM1 CDC3 CDC10 BUD20 SHS1 RVS161 SEC15 CDC11 CDC12 TWF1 PEF1
GO:0044248 cellular catabolic process	1,87E-004	50/245 (20.4%)	785/6346 (12.4%)	BNA1 BRO1 GAL7 ISC1 CHA1 LAP3 VID24 TFB3 GGA2 CPS1 GAT1 ATH1 DUG1 CDD1 ATG2 PEP1 STO1 PHO8 ECI1 RRP46 RRP42 NPR2 TPA1 PNP1 PPX1 DCI1 APN2 DAL2 CSF1 GSH1 AIR2 NTE1 HEM14 JHD1 ARG82 NUC1 VAM6 VID30 ARO9 NAS2 DUG2 YEA4 PRB1 DAN4 BAR1 YNR065C YMR034C VPS8 RAD14 PEF1
GO:0007121 bipolar cellular bud site selection	2,58E-004	7/245 (2.9%)	34/6346 (0.5%)	END3 CDC10 RVS161 SEC15 CDC12 TWF1 PEF1
GO:0000910 cytokinesis	2,89E-004	13/245 (5.3%)	110/6346 (1.7%)	END3 ELM1 CDC3 CDC10 BUD20 SHS1 IQG1 RVS161 SEC15 CDC11 CDC12 TWF1 PEF1
GO:0006623 protein targeting to vacuole	5,57E-004	9/245 (3.7%)	62/6346 (1.0%)	BRO1 VID24 VPS27 GGA2 MVP1 ATG2 PEP1 VPS1 HSE1
GO:0019363 pyridine nucleotide biosynthetic process	5,77E-004	4/245 (1.6%)	11/6346 (0.2%)	BNA1 BNA5 BNA4 BNA2
GO:0051171 regulation of nitrogen compound metabolic process	5,77E-004	4/245 (1.6%)	11/6346 (0.2%)	GAT1 GZF3 ARG82 VID30
GO:0016192 vesicle-mediated transport	7,38E-004	26/245 (10.6%)	345/6346 (5.4%)	BRO1 GRH1 ERV29 END3 VID24 VPS27 VPS60 GGA2 AVL9 SVP26 THR4 ERP4 ENT4 BET5 RVS161 SRO7 BZZ1 LAA1 VTH1 SEC15 VTH2 VPS8 HSE1 VPS74 SNX3 PEP12
GO:0046907 intracellular transport	8,93E-004	38/245 (15.5%)	586/6346 (9.2%)	BRO1 GRH1 ERV29 TIM13 VID24 VPS27 VPS60 GGA2 AVL9 MVP1 ATG2 PEP1 SVP26 ERP4 BET5 MIA40 AIR2 SAM50 SRO7 LAA1 VTH1 VPS1 VAM6 SEC15 NUP157 DYN1 PCP1 TOM70 NUP84 VTH2 PEX13 VPS8 HSE1 VPS74 SNX3 SEC62 UPS1 PEP12
GO:0006605 protein targeting	1,34E-003	19/245 (7.8%)	230/6346 (3.6%)	BRO1 TIM13 VID24 VPS27 GGA2 MVP1 ATG2 PEP1 MIA40 SAM50 VPS1 NUP157 PCP1 TOM70 NUP84 PEX13 HSE1 SEC62 UPS1
GO:0048869 cellular developmental process	1,36E-003	23/245 (9.4%)	303/6346 (4.8%)	SPR3 PXL1 END3 EMI5 RSC1 ELM1 CDC3 CDC10 FKH1 BUD20 SHS1 SPT3 RVS161 SRO7 SEC15 CDC11 CDC12 PRB1 TWF1 SPR28 RGA1 LRG1 PEF1
GO:0034067 protein localization in Golgi apparatus	1,60E-003	4/245 (1.6%)	14/6346 (0.2%)	VPS27 SVP26 VPS1 VPS74
GO:0051188 cofactor biosynthetic process	1,79E-003	10/245 (4.1%)	87/6346 (1.4%)	BNA1 BNA5 ISD11 GSH1 JAC1 HEM14 BNA4 BNA2 CAT5 FAU1
GO:0051649 establishment of localization in cell	1,88E-003	39/245 (15.9%)	631/6346 (9.9%)	BUB1 BRO1 GRH1 ERV29 TIM13 VID24 VPS27 VPS60 GGA2 AVL9 MVP1 ATG2 PEP1 SVP26 ERP4 BET5 MIA40 AIR2 SAM50 SRO7 LAA1 VTH1 VPS1 VAM6 SEC15 NUP157 DYN1 PCP1 TOM70 NUP84 VTH2 PEX13 VPS8 HSE1 VPS74 SNX3 SEC62 UPS1 PEP12
GO:0045324 late endosome to vacuole transport	1,97E-003	6/245 (2.4%)	35/6346 (0.6%)	BRO1 VPS27 VPS60 GGA2 VPS8 HSE1
GO:0033365 protein localization in organelle	2,11E-003	4/245 (1.6%)	15/6346 (0.2%)	VPS27 SVP26 VPS1 VPS74
GO:0006493 protein amino acid O-linked glycosylation	2,11E-003	4/245 (1.6%)	15/6346 (0.2%)	PMT2 PMT4 PMT6 PMT3
GO:0016197 endosome transport	2,20E-003	8/245 (3.3%)	61/6346 (1.0%)	BRO1 VPS27 VPS60 GGA2 LAA1 VPS8 HSE1 SNX3
GO:0006914 autophagy	2,66E-003	19/245 (7.8%)	244/6346 (3.8%)	ISC1 TFB3 ATG2 PEP1 PHO8 NPR2 APN2 GSH1 HEM14 JHD1 ARG82 VAM6 ARO9 YEA4 YNR065C YMR034C VPS8 RAD14 PEF1
GO:0000747 conjugation with cellular fusion	2,68E-003	12/245 (4.9%)	123/6346 (1.9%)	SPR3 CDC3 SCP160 CSN12 CDC10 SHS1 SPT3 FAR11 CDC11 CDC12 BAR1 RGA1
GO:0005978 glycogen biosynthetic process	2,73E-003	4/245 (1.6%)	16/6346 (0.3%)	GSY2 GLG2 PIG2 GSY1
GO:0008104 protein localization	2,97E-003	24/245 (9.8%)	341/6346 (5.4%)	BRO1 TIM13 VID24 VPS27 GGA2 MVP1 ATG2 PEP1 SVP26 MIA40 SAM50 LAA1 VPS1 NUP157 PCP1 TOM70 TWF1 NUP84 PEX13 HSE1 VPS74 SNX3 SEC62 UPS1
GO:0000746 conjugation	3,07E-003	12/245 (4.9%)	125/6346 (2.0%)	SPR3 CDC3 SCP160 CSN12 CDC10 SHS1 SPT3 FAR11 CDC11 CDC12 BAR1 RGA1
GO:0019953 sexual reproduction	3,07E-003	12/245 (4.9%)	125/6346 (2.0%)	SPR3 CDC3 SCP160 CSN12 CDC10 SHS1 SPT3 FAR11 CDC11 CDC12 BAR1 RGA1
GO:0006886 intracellular protein transport	3,65E-003	19/245 (7.8%)	251/6346 (4.0%)	BRO1 TIM13 VID24 VPS27 GGA2 MVP1 ATG2 PEP1 MIA40 SAM50 VPS1 NUP157 PCP1 TOM70 NUP84 PEX13 HSE1 SEC62 UPS1
GO:0009108 coenzyme biosynthetic process	4,41E-003	7/245 (2.9%)	54/6346 (0.9%)	BNA1 BNA5 GSH1 BNA4 BNA2 CAT5 FAU1
GO:0051641 cellular localization	5,19E-003	40/245 (16.3%)	690/6346 (10.9%)	BUB1 BRO1 GRH1 ERV29 TIM13 VID24 VPS27 VPS60 GGA2 AVL9 MVP1 SCP160 ATG2 PEP1 SVP26 ERP4 BET5 MIA40 AIR2 SAM50 SRO7 LAA1 VTH1 VPS1 VAM6 SEC15 NUP157 DYN1 PCP1 TOM70 NUP84 VTH2 PEX13 VPS8 HSE1 VPS74 SNX3 SEC62 UPS1 PEP12
GO:0032502 developmental process	5,66E-003	26/245 (10.6%)	399/6346 (6.3%)	SPR3 PXL1 END3 PDE2 EMI5 RSC1 ELM1 CDC3 CDC10 FKH1 BUD20 SHS1 SPT3 RVS161 SRO7 SCP1 NUC1 SEC15 CDC11 CDC12 PRB1 TWF1 SPR28 RGA1 LRG1 PEF1
GO:0007039 vacuolar protein catabolic process	6,40E-003	11/245 (4.5%)	120/6346 (1.9%)	BNA1 VID24 CPS1 GAT1 DUG1 ATG2 CSF1 VAM6 VID30 PRB1 DAN4
GO:0044242 cellular lipid catabolic process	6,44E-003	4/245 (1.6%)	20/6346 (0.3%)	ISC1 ECI1 DCI1 NTE1
GO:0016042 lipid catabolic process	6,44E-003	4/245 (1.6%)	20/6346 (0.3%)	ISC1 ECI1 DCI1 NTE1
GO:0005977 glycogen metabolic process	7,06E-003	5/245 (2.0%)	32/6346 (0.5%)	GSY2 GLG2 PIG2 GSY1 GIP2
GO:0033036 macromolecule localization	7,08E-003	26/245 (10.6%)	406/6346 (6.4%)	BRO1 TIM13 VID24 VPS27 GGA2 MVP1 SCP160 ATG2 PEP1 SVP26 MIA40 AIR2 SAM50 LAA1 VPS1 NUP157 PCP1 TOM70 TWF1 NUP84 PEX13 HSE1 VPS74 SNX3 SEC62 UPS1
GO:0015031 protein transport	7,08E-003	19/245 (7.8%)	267/6346 (4.2%)	BRO1 TIM13 VID24 VPS27 GGA2 MVP1 ATG2 PEP1 MIA40 SAM50 VPS1 NUP157 PCP1 TOM70 NUP84 PEX13 HSE1 SEC62 UPS1
GO:0044255 cellular lipid metabolic process	7,39E-003	17/245 (6.9%)	230/6346 (3.6%)	ISC1 OAR1 PGS1 OPI3 TSC10 SMP3 BTS1 FAT1 ERG2 ERG27 ECI1 DCI1 NTE1 ARG82 CWH43 TSC11 TES1
GO:0030036 actin cytoskeleton organization	7,52E-003	10/245 (4.1%)	106/6346 (1.7%)	END3 ENT4 IQG1 BZZ1 SCP1 VPS1 PIN3 TWF1 TSC11 RGA1
GO:0019674 NAD metabolic process	7,72E-003	4/245 (1.6%)	21/6346 (0.3%)	BNA1 BNA5 BNA4 BNA2
GO:0032505 reproduction of a single-celled organism	7,96E-003	16/245 (6.5%)	213/6346 (3.4%)	SPR3 END3 EMI5 ELM1 CDC3 CDC10 FKH1 SHS1 SPT3 RVS161 SEC15 CDC11 CDC12 TWF1 RGA1 PEF1
GO:0051704 multi-organism process	8,06E-003	12/245 (4.9%)	141/6346 (2.2%)	SPR3 CDC3 SCP160 CSN12 CDC10 SHS1 SPT3 FAR11 CDC11 CDC12 BAR1 RGA1
GO:0045185 maintenance of protein location	8,06E-003	5/245 (2.0%)	33/6346 (0.5%)	VPS27 PEP1 SVP26 VPS1 TWF1
