# Supplementary material for: Evolutionary analysis of the ENTH/ANTH/VHS protein superfamily reveals a coevolution between membrane trafficking and metabolism
Source: BMC Genomics. 2012 Jul 2;13:297. doi: 10.1186/1471-2164-13-297 (PMC3473312; doi:10.1186/1471-2164-13-297)
Supplement: Additional file 5 — Figure S5. GO Term Enrichment of yeast-plant and yeast-protist proteins.The complete analysis of the yeast S. cerevisiae GO Term Enrichment analysis for cellular processes is shown, only categories with a P-value inferior to 10-3 and comprising more than 3 proteins were retained. (A) Proteins common to S. cerevisiae and A. thaliana are all involved in metabolism (mainly in amino-acids and some vitamins biosynthesis pathways) and highlighted in green. (B) Proteins common to S. cerevisiae and E. histolytica are involved in the chitin metabolism. Chitin is a major component of the yeast cell wall and of the Entamoeba cyst wall. (PDF 426 kb) [file 1471-2164-13-297-S5.pdf]

Biological process between *S. cerevisiae* and *A. thaliana*

GO Term	P-value	Sample freq	Background freq	Genes
GO:0044283 small molecule biosynthetic process	8.74e-20	70/277 (25.3%)	439/6357 (6.9%)	HIS7 URA1 THI3 HIS5 ILV5 PHA2 BIO2 TRP1 SNO3 THI22 PAN6 MET16 TRP2 MHT1 HOM6 LYS20 ERG6 HOM2 FOL1 SAM4 ADE1 LEU9 ADE2 MET1 ILV6 ARG7 THI6 ADH2 LYS21 MTD1 GLY1 TRP3 HOM3 ARO7 PCK1 SNZ1 ARO2 ERG8 URA4 RIB3 SNO2 TRS31 RIB1 HIS1 ABZ1 ILV3 HIS3 TRP4 HIS6 CTR86 THI4 FYV10 MET6 ARO10 SNO1 ARO1 RIB5 RIB4 SNZ3 ECM31 THI21 SUR4 TRP5 ADH1 FMS1 LEU4 SNZ2 THI20 HIS4 URH1
GO:0044271 cellular nitrogen compound biosynthetic process	4.57e-19	61/277 (22.0%)	346/6357 (5.4%)	HIS7 URA1 THI3 HIS5 ILV5 PHA2 BIO2 TRP1 SNO3 THI22 PAN6 MET16 TRP2 MHT1 HOM6 LYS20 HOM2 FOL1 SAM4 ADE1 LEU9 ADE2 MET1 ILV6 ARG7 THI6 RMA1 LYS21 MTD1 GLY1 TRP3 HOM3 ARO7 ARO2 URA4 RIB3 SNO2 TRS31 RIB1 HIS1 ABZ1 ILV3 HIS3 TRP4 HIS6 CTR86 THI4 MET6 ARO1 RIB5 RIB4 SNZ3 ECM31 THI21 TRP5 FMS1 LEU4 SNZ2 THI20 HIS4 URH1
GO:0044281 small molecule metabolic process	3.26e-18	105/277 (37.9%)	964/6357 (15.2%)	HIS7 URA1 THI3 HIS5 ILV5 RMT2 PHA2 BIO2 YLR126C TRP1 SNO3 THI22 GTO1 MLS1 PAN6 MET16 TRP2 SDT1 MHT1 HOM6 LYS20 ADH7 ERG6 HOM2 FOL1 DAL4 SAM4 ADE1 YOR283W LEU9 ADE2 MET1 ILV6 PCL6 ARG7 THI6 PDR16 ADH2 RMA1 LYS21 MTD1 GLY1 TRP3 ECM4 ICL1 YGL039W HOM3 ARO7 ADH6 ATG26 RKM3 TGL4 PCK1 SNZ1 ARO2 ERG8 URA4 GRE2 RIB3 SNO2 TRS31 RIB1 HIS1 ABZ1 ILV3 HIS3 PCL7 DUR3 TRP4 NDE1 TGL5 HIS6 YMR315W NDI1 CTR86 THI4 FYV10 MET6 ARO10 SNO1 ARO1 RIB5 LRO1 RIB4 SNZ3 ECM31 TGL3 NDE2 ICL2 THI21 SUR4 TRP5 GTO3 SWA2 ADH1 FMS1 LEU4 YJU3 PSD2 PCD1 SNZ2 THI20 HIS4 DAL7 URH1
GO:0019438 aromatic compound biosynthetic process	1.61e-17	23/277 (8.3%)	43/6357 (0.7%)	THI3 PHA2 TRP1 SNO3 THI22 TRP2 FOL1 THI6 RMA1 MTD1 TRP3 ARO7 ARO2 SNO2 ABZ1 TRP4 THI4 ARO1 SNZ3 THI21 TRP5 SNZ2 THI20
GO:0006725 cellular aromatic compound metabolic process	9.05e-17	31/277 (11.2%)	93/6357 (1.5%)	URA1 THI3 PHA2 TRP1 SNO3 THI22 TRP2 SDT1 FOL1 DAL4 ADE2 THI6 RMA1 MTD1 TRP3 YGL039W ARO7 ARO2 URA4 SNO2 ABZ1 TRP4 THI4 ARO10 ARO1 SNZ3 THI21 TRP5 SNZ2 THI20 DAL7
GO:0008652 cellular amino acid biosynthetic process	5.91e-16	38/277 (13.7%)	154/6357 (2.4%)	HIS7 HIS5 ILV5 PHA2 TRP1 PAN6 MET16 TRP2 MHT1 HOM6 LYS20 HOM2 FOL1 SAM4 LEU9 MET1 ILV6 ARG7 LYS21 GLY1 TRP3 HOM3 ARO7 ARO2 HIS1 ABZ1 ILV3 HIS3 TRP4 HIS6 CTR86 MET6 ARO1 ECM31 TRP5 FMS1 LEU4 HIS4
GO:0009309 amine biosynthetic process	1.54e-15	38/277 (13.7%)	158/6357 (2.5%)	HIS7 HIS5 ILV5 PHA2 TRP1 PAN6 MET16 TRP2 MHT1 HOM6 LYS20 HOM2 FOL1 SAM4 LEU9 MET1 ILV6 ARG7 LYS21 GLY1 TRP3 HOM3 ARO7 ARO2 HIS1 ABZ1 ILV3 HIS3 TRP4 HIS6 CTR86 MET6 ARO1 ECM31 TRP5 FMS1 LEU4 HIS4
GO:0009308 amine metabolic process	1.56e-15	55/277 (19.9%)	332/6357 (5.2%)	HIS7 THI3 HIS5 ILV5 PHA2 YLR126C TRP1 SNO3 GTO1 PAN6 MET16 TRP2 MHT1 HOM6 LYS20 CTS1 HOM2 FOL1 DAL4 SAM4 LEU9 MET1 ILV6 ARG7 ADH2 LYS21 GLY1 TRP3 ECM4 HOM3 ARO7 ARO2 SNO2 HIS1 ABZ1 ILV3 HIS3 TRP4 HIS6 CTR86 MET6 ARO10 SNO1 ARO1 ECM31 ICL2 TRP5 GTO3 CRH1 ADH1 FMS1 LEU4 PSD2 HIS4 DAL7
GO:0006520 cellular amino acid metabolic process	8.55e-15	50/277 (18.1%)	287/6357 (4.5%)	HIS7 THI3 HIS5 ILV5 PHA2 YLR126C TRP1 SNO3 GTO1 PAN6 MET16 TRP2 MHT1 HOM6 LYS20 HOM2 FOL1 SAM4 LEU9 MET1 ILV6 ARG7 ADH2 LYS21 GLY1 TRP3 ECM4 HOM3 ARO7 ARO2 SNO2 HIS1 ABZ1 ILV3 HIS3 TRP4 HIS6 CTR86 MET6 ARO10 SNO1 ARO1 ECM31 ICL2 TRP5 GTO3 ADH1 FMS1 LEU4 HIS4
GO:0044106 cellular amine metabolic process	1.52e-14	51/277 (18.4%)	302/6357 (4.8%)	HIS7 THI3 HIS5 ILV5 PHA2 YLR126C TRP1 SNO3 GTO1 PAN6 MET16 TRP2 MHT1 HOM6 LYS20 HOM2 FOL1 SAM4 LEU9 MET1 ILV6 ARG7 ADH2 LYS21 GLY1 TRP3 ECM4 HOM3 ARO7 ARO2 SNO2 HIS1 ABZ1 ILV3 HIS3 TRP4 HIS6 CTR86 MET6 ARO10 SNO1 ARO1 ECM31 ICL2 TRP5 GTO3 ADH1 FMS1 LEU4 PSD2 HIS4
GO:0046483 heterocycle metabolic process	4.77e-14	54/277 (19.5%)	345/6357 (5.4%)	HIS7 URA1 THI3 HIS5 BIO2 TRP1 SNO3 THI22 TRP2 SDT1 FOL1 DAL4 SAM4 ADE1 ADE2 MET1 THI6 ADH2 ARA2 RMA1 MTD1 TRP3 ADH6 SNZ1 URA4 RIB3 SNO2 TRS31 RIB1 HIS1 ABZ1 HIS3 TRP4 NDE1 HIS6 ALO1 YMR315W NDI1 THI4 ARO10 SNO1 RIB5 RIB4 SNZ3 NDE2 THI21 TRP5 SWA2 ADH1 SNZ2 THI20 HIS4 DAL7 URH1
GO:0016053 organic acid biosynthetic process	2.77e-13	40/277 (14.4%)	202/6357 (3.2%)	HIS7 HIS5 ILV5 PHA2 BIO2 TRP1 PAN6 MET16 TRP2 MHT1 HOM6 LYS20 HOM2 FOL1 SAM4 LEU9 MET1 ILV6 ARG7 LYS21 GLY1 TRP3 HOM3 ARO7 ARO2 HIS1 ABZ1 ILV3 HIS3 TRP4 HIS6 CTR86 MET6 ARO1 ECM31 SUR4 TRP5 FMS1 LEU4 HIS4
GO:0046394 carboxylic acid biosynthetic process	2.77e-13	40/277 (14.4%)	202/6357 (3.2%)	HIS7 HIS5 ILV5 PHA2 BIO2 TRP1 PAN6 MET16 TRP2 MHT1 HOM6 LYS20 HOM2 FOL1 SAM4 LEU9 MET1 ILV6 ARG7 LYS21 GLY1 TRP3 HOM3 ARO7 ARO2 HIS1 ABZ1 ILV3 HIS3 TRP4 HIS6 CTR86 MET6 ARO1 ECM31 SUR4 TRP5 FMS1 LEU4 HIS4
GO:0006082 organic acid metabolic process	2.48e-12	56/277 (20.2%)	402/6357 (6.3%)	HIS7 THI3 HIS5 ILV5 PHA2 BIO2 YLR126C TRP1 SNO3 GTO1 MLS1 PAN6 MET16 TRP2 MHT1 HOM6 LYS20 HOM2 FOL1 SAM4 LEU9 MET1 ILV6 ARG7 ADH2 LYS21 GLY1 TRP3 ECM4 ICL1 HOM3 ARO7 ARO2 ERG8 SNO2 HIS1 ABZ1 ILV3 HIS3 TRP4 HIS6 CTR86 MET6 ARO10 SNO1 ARO1 ECM31 ICL2 SUR4 TRP5 GTO3 ADH1 FMS1 LEU4 HIS4 DAL7
GO:0018130 heterocycle biosynthetic process	3.41e-12	39/277 (14.1%)	206/6357 (3.2%)	HIS7 URA1 THI3 HIS5 BIO2 TRP1 SNO3 THI22 TRP2 FOL1 ADE1 ADE2 MET1 THI6 ARA2 RMA1 MTD1 TRP3 URA4 RIB3 SNO2 TRS31 RIB1 HIS1 ABZ1 HIS3 TRP4 HIS6 ALO1 THI4 RIB5 RIB4 SNZ3 THI21 TRP5 SNZ2 THI20 HIS4 URH1
GO:0043436 oxoacid metabolic process	9.34e-12	55/277 (19.9%)	401/6357 (6.3%)	HIS7 THI3 HIS5 ILV5 PHA2 BIO2 YLR126C TRP1 SNO3 GTO1 MLS1 PAN6 MET16 TRP2 MHT1 HOM6 LYS20 HOM2 FOL1 SAM4 LEU9 MET1 ILV6 ARG7 ADH2 LYS21 GLY1 TRP3 ECM4 ICL1 HOM3 ARO7 ARO2 SNO2 HIS1 ABZ1 ILV3 HIS3 TRP4 HIS6 CTR86 MET6 ARO10 SNO1 ARO1 ECM31 ICL2 SUR4 TRP5 GTO3 ADH1 FMS1 LEU4 HIS4 DAL7
GO:0019752 carboxylic acid metabolic process	9.34e-12	55/277 (19.9%)	401/6357 (6.3%)	HIS7 THI3 HIS5 ILV5 PHA2 BIO2 YLR126C TRP1 SNO3 GTO1 MLS1 PAN6 MET16 TRP2 MHT1 HOM6 LYS20 HOM2 FOL1 SAM4 LEU9 MET1 ILV6 ARG7 ADH2 LYS21 GLY1 TRP3 ECM4 ICL1 HOM3 ARO7 ARO2 SNO2 HIS1 ABZ1 ILV3 HIS3 TRP4 HIS6 CTR86 MET6 ARO10 SNO1 ARO1 ECM31 ICL2 SUR4 TRP5 GTO3 ADH1 FMS1 LEU4 HIS4 DAL7
GO:0042180 cellular ketone metabolic process	1.41e-11	57/277 (20.6%)	431/6357 (6.8%)	HIS7 THI3 HIS5 ILV5 PHA2 BIO2 YLR126C TRP1 SNO3 GTO1 MLS1 PAN6 MET16 TRP2 MHT1 HOM6 LYS20 HOM2 FOL1 SAM4 LEU9 MET1 ILV6 ARG7 ADH2 LYS21 GLY1 TRP3 ECM4 ICL1 YGL039W HOM3 ARO7 ARO2 SNO2 HIS1 ABZ1 ILV3 HIS3 TRP4 HIS6 CTR86 FYV10 MET6 ARO10 SNO1 ARO1 ECM31 ICL2 SUR4 TRP5 GTO3 ADH1 FMS1 LEU4 HIS4 DAL7
GO:0009110 vitamin biosynthetic process	2.93e-11	23/277 (8.3%)	73/6357 (1.1%)	THI3 BIO2 SNO3 THI22 PAN6 FOL1 THI6 SNZ1 RIB3 SNO2 RIB1 ABZ1 THI4 SNO1 RIB5 RIB4 SNZ3 ECM31 THI21 FMS1 SNZ2 THI20 URH1
GO:0042364 water-soluble vitamin biosynthetic process	2.93e-11	23/277 (8.3%)	73/6357 (1.1%)	THI3 BIO2 SNO3 THI22 PAN6 FOL1 THI6 SNZ1 RIB3 SNO2 RIB1 ABZ1 THI4 SNO1 RIB5 RIB4 SNZ3 ECM31 THI21 FMS1 SNZ2 THI20 URH1
GO:0006767 water-soluble vitamin metabolic process	5.67e-11	23/277 (8.3%)	75/6357 (1.2%)	THI3 BIO2 SNO3 THI22 PAN6 FOL1 THI6 SNZ1 RIB3 SNO2 RIB1 ABZ1 THI4 SNO1 RIB5 RIB4 SNZ3 ECM31 THI21 FMS1 SNZ2 THI20 URH1
GO:0006766 vitamin metabolic process	5.67e-11	23/277 (8.3%)	75/6357 (1.2%)	THI3 BIO2 SNO3 THI22 PAN6 FOL1 THI6 SNZ1 RIB3 SNO2 RIB1 ABZ1 THI4 SNO1 RIB5 RIB4 SNZ3 ECM31 THI21 FMS1 SNZ2 THI20 URH1
GO:0006790 sulfur compound metabolic process	2.29e-08	24/277 (8.7%)	106/6357 (1.7%)	THI3 BIO2 OPT1 SNO3 THI22 GTO1 MET16 MHT1 HOM6 HOM2 SAM4 MET1 THI6 ECM4 HOM3 SNO2 THI4 MET6 ARO10 SNZ3 THI21 GTO3 SNZ2 THI20
GO:0009073 aromatic amino acid family biosynthetic process	1.75e-07	9/277 (3.2%)	12/6357 (0.2%)	PHA2 TRP1 TRP2 TRP3 ARO7 ARO2 TRP4 ARO1 TRP5
GO:0046417 chorismate metabolic process	1.75e-07	9/277 (3.2%)	12/6357 (0.2%)	PHA2 TRP1 TRP2 TRP3 ARO7 ARO2 TRP4 ARO1 TRP5
GO:0009228 thiamine biosynthetic process	5.60e-07	10/277 (3.6%)	17/6357 (0.3%)	THI3 SNO3 THI22 THI6 SNO2 THI4 SNZ3 THI21 SNZ2 THI20
GO:0009072 aromatic amino acid family metabolic process	7.29e-07	11/277 (4.0%)	22/6357 (0.3%)	PHA2 TRP1 TRP2 TRP3 ARO7 ARO2 ABZ1 TRP4 ARO10 ARO1 TRP5
GO:0042724 thiamine-containing compound biosynthetic process	1.21e-06	10/277 (3.6%)	18/6357 (0.3%)	THI3 SNO3 THI22 THI6 SNO2 THI4 SNZ3 THI21 SNZ2 THI20
GO:0044272 sulfur compound biosynthetic process	2.10e-06	18/277 (6.5%)	73/6357 (1.1%)	THI3 BIO2 SNO3 THI22 MET16 MHT1 HOM6 HOM2 SAM4 MET1 THI6 SNO2 THI4 MET6 SNZ3 THI21 SNZ2 THI20
GO:0006772 thiamine metabolic process	2.46e-06	10/277 (3.6%)	19/6357 (0.3%)	THI3 SNO3 THI22 THI6 SNO2 THI4 SNZ3 THI21 SNZ2 THI20
GO:0042723 thiamine-containing compound metabolic process	4.73e-06	10/277 (3.6%)	20/6357 (0.3%)	THI3 SNO3 THI22 THI6 SNO2 THI4 SNZ3 THI21 SNZ2 THI20
GO:0055114 oxidation-reduction process	7.59e-06	46/277 (16.6%)	424/6357 (6.7%)	URA1 GRX1 ILV5 MLS1 AHP1 MET16 FET3 HOM6 ADH7 HOM2 CCS1 FET5 MET1 PCL6 ADH2 ARA2 MTD1 ICL1 YGL039W YDR541C ADH6 PCK1 OYE3 FRE6 GRE2 AIF1 QCR2 YML131W RIB3 PCL7 NDE1 CCP1 ALO1 DOT5 YMR315W NDI1 ARI1 YKL071W ARO1 NDE2 ADH1 FMS1 YPR127W HIS4 DAL7 OYE2
GO:0042435 indole-containing compound biosynthetic process	2.72e-04	5/277 (1.8%)	5/6357 (0.1%)	TRP1 TRP2 TRP3 TRP4 TRP5
GO:0046219 indolalkylamine biosynthetic process	2.72e-04	5/277 (1.8%)	5/6357 (0.1%)	TRP1 TRP2 TRP3 TRP4 TRP5
GO:0000162 tryptophan biosynthetic process	2.72e-04	5/277 (1.8%)	5/6357 (0.1%)	TRP1 TRP2 TRP3 TRP4 TRP5
GO:0043648 dicarboxylic acid metabolic process	4.26e-04	9/277 (3.2%)	23/6357 (0.4%)	PHA2 TRP1 TRP2 TRP3 ARO7 ARO2 TRP4 ARO1 TRP5
GO:0009066 aspartate family amino acid metabolic process	9.47e-04	14/277 (5.1%)	65/6357 (1.0%)	MET16 MHT1 HOM6 LYS20 HOM2 SAM4 MET1 LYS21 GLY1 HOM3 CTR86 MET6 ARO10 ICL2
GO:0006566 threonine metabolic process	2.11e-03	6/277 (2.2%)	10/6357 (0.2%)	HOM6 HOM2 GLY1 HOM3 CTR86 ICL2
GO:0008615 pyridoxine biosynthetic process	2.11e-03	6/277 (2.2%)	10/6357 (0.2%)	SNO3 SNZ1 SNO2 SNO1 SNZ3 SNZ2
GO:0008614 pyridoxine metabolic process	2.11e-03	6/277 (2.2%)	10/6357 (0.2%)	SNO3 SNZ1 SNO2 SNO1 SNZ3 SNZ2
GO:0042816 vitamin B6 metabolic process	2.11e-03	6/277 (2.2%)	10/6357 (0.2%)	SNO3 SNZ1 SNO2 SNO1 SNZ3 SNZ2
GO:0042819 vitamin B6 biosynthetic process	2.11e-03	6/277 (2.2%)	10/6357 (0.2%)	SNO3 SNZ1 SNO2 SNO1 SNZ3 SNZ2
GO:0009075 histidine family amino acid metabolic process	4.47e-03	6/277 (2.2%)	11/6357 (0.2%)	HIS7 HIS5 HIS1 HIS3 HIS6 HIS4
GO:0000105 histidine biosynthetic process	4.47e-03	6/277 (2.2%)	11/6357 (0.2%)	HIS7 HIS5 HIS1 HIS3 HIS6 HIS4
GO:0006547 histidine metabolic process	4.47e-03	6/277 (2.2%)	11/6357 (0.2%)	HIS7 HIS5 HIS1 HIS3 HIS6 HIS4
GO:0009076 histidine family amino acid biosynthetic process	4.47e-03	6/277 (2.2%)	11/6357 (0.2%)	HIS7 HIS5 HIS1 HIS3 HIS6 HIS4
GO:0009081 branched chain family amino acid metabolic process	5.84e-03	8/277 (2.9%)	23/6357 (0.4%)	THI3 ILV5 HOM6 LEU9 ILV6 ILV3 ARO10 LEU4
GO:0042430 indole-containing compound metabolic process	8.61e-03	6/277 (2.2%)	12/6357 (0.2%)	TRP1 TRP2 TRP3 TRP4 ARO10 TRP5
GO:0006568 tryptophan metabolic process	8.61e-03	6/277 (2.2%)	12/6357 (0.2%)	TRP1 TRP2 TRP3 TRP4 ARO10 TRP5
GO:0006586 indolalkylamine metabolic process	8.61e-03	6/277 (2.2%)	12/6357 (0.2%)	TRP1 TRP2 TRP3 TRP4 ARO10 TRP5
GO:0030001 metal ion transport	9.89e-03	15/277 (5.4%)	89/6357 (1.4%)	VCX1 MMT1 VNX1 KHA1 FET3 CCS1 FET5 FRE6 ZRT2 DUR3 ZRT1 CCC1 MMT2 PHO91 ZRT3

Biological process between *S. cerevisiae* and *E. histolitica*

GO Term	Aspect	P-value	Sample frequency	Background frequency	Genes
GO:0006023 aminoglycan biosynthetic process	P	7.91e-03	3/20 (15.0%)	16/6357 (0.3%)	SKT5 CHS1 CHS3
GO:0006031 chitin biosynthetic process	P	7.91e-03	3/20 (15.0%)	16/6357 (0.3%)	SKT5 CHS1 CHS3
