# Supplementary material for: Evolutionary analysis of the ENTH/ANTH/VHS protein superfamily reveals a coevolution between membrane trafficking and metabolism
Source: BMC Genomics. 2012 Jul 2;13:297. doi: 10.1186/1471-2164-13-297 (PMC3473312; doi:10.1186/1471-2164-13-297)
Supplement: Additional file 2 — Figure S2. Aligment of the VHS domains of GGA and TOM proteins. (A) The sequences of the VHS domain of Metazoa, Fungi, Plantae and Prostists GGA were aligned and the secondary structure of human GGA1 is shown above the sequence alignment (PDB ID code 1JWF). (B) The sequences of the VHS domain of Metazoa TOM were aligned and the secondary structure of human TOM1 is shown above the sequence alignment (PDB ID code 1ELK). Arrows indicate features specific to the TOM family. ESPript website was used for displaying purposes [60]. [file 1471-2164-13-297-S2.pdf]

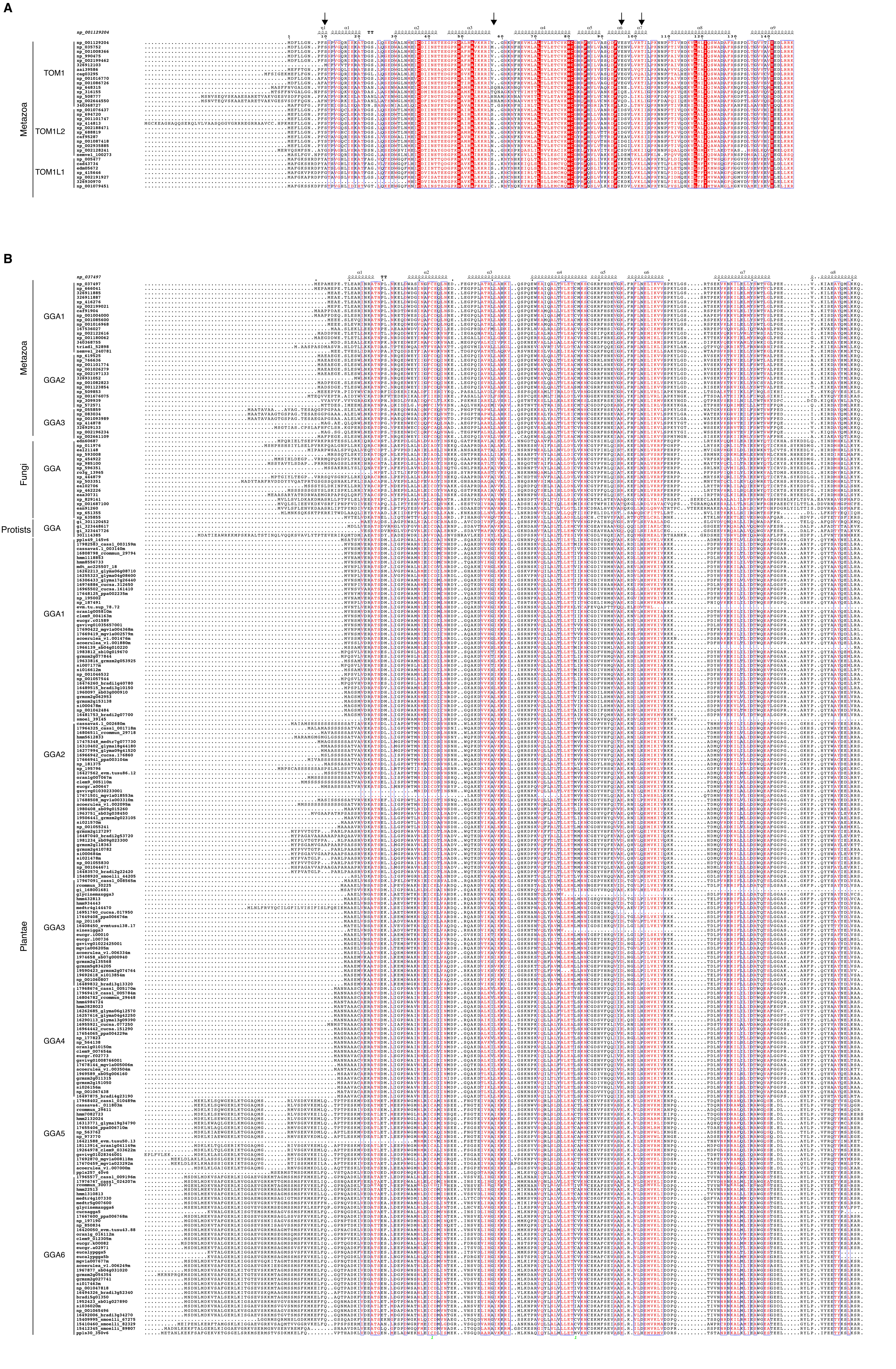
A
Metazoa
TOM1
TOM1L2
TOM1L1
B
Metazoa
GGA1
GGA2
GGA3
Fungi
GGA
Protists
GGA
Plantae
GGA1
GGA2
GGA3
GGA4
GGA5
GGA6
